# Methylation Affects Transposition and Splicing of a Large CACTA Transposon from a MYB Transcription Factor Regulating Anthocyanin Synthase Genes in Soybean Seed Coats

**DOI:** 10.1371/journal.pone.0111959

**Published:** 2014-11-04

**Authors:** Gracia Zabala, Lila O. Vodkin

**Affiliations:** Department of Crop Sciences, University of Illinois, Urbana, Illinois, United States of America; Institute of Hydrobiology, Chinese Academy of Sciences, China

## Abstract

We determined the molecular basis of three soybean lines that vary in seed coat color at the *R* locus which is thought to encode a MYB transcription factor. RM55-*r^m^* is homozygous for a mutable allele (*r^m^*) that specifies black and brown striped seeds; RM30-R* is a stable black revertant isoline derived from the mutable line; and RM38-*r* has brown seed coats due to a recessive *r* allele shown to translate a truncated MYB protein. Using long range PCR, 454 sequencing of amplicons, and whole genome re-sequencing, we determined that the variegated RM55-*r^m^* line had a 13 kb CACTA subfamily transposon insertion (designated TgmR*) at a position 110 bp from the beginning of Intron2 of the *R* locus, Glyma09g36983. Although the MYB encoded by *R* was expressed at only very low levels in older seed coats of the black revertant RM30-R* line, it upregulated expression of anthocyanidin synthase genes (*ANS2, ANS3*) to promote the synthesis of anthocyanins. Surprisingly, the RM30-R* revertant also carried the 13 kb TgmR* insertion in Intron2. Using RNA-Seq, we showed that intron splicing was accurate, albeit at lower levels, despite the presence of the 13 kb TgmR* element. As determined by whole genome methylation sequencing, we demonstrate that the TgmR* sequence was relatively more methylated in RM30-R* than in the mutable RM55-*r^m^* progenitor line. The stabilized and more methylated RM30-R* revertant line apparently lacks effective binding of a transposae to its subterminal repeats, thus allowing intron splicing to proceed resulting in sufficient MYB protein to stimulate anthocyanin production and thus black seed coats. In this regard, the TgmR* element in soybean resembles McClintock's *Spm-*suppressible and change-of-state alleles of maize. This comparison explains the opposite effects of the TgmR* element on intron splicing of the MYB gene in which it resides depending on the methylation state of the element.

## Introduction

Anthocyanins are end products of three branches of the flavonoid pathway with important functions in plant defense against pathogens and protection from UV light. Because of their antioxidant properties, seeds and vegetables with anthocyanin pigments have added nutritional and health value. Understanding the regulation and expression of all regulatory genes in the anthocyanin metabolic pathway in each major crop plant species is of significance as a model system for gene expression and for improving agronomic and nutritional properties.

We have identified the molecular basis of some of the classical loci leading to anthocyanin production in soybean (*Glycine max*) seed and plant parts. The majority of the cultivated soybean varieties have yellow seeds with hila of various colors but ancestral soybeans (*Glycine soja*) have pigmented seed coats. The pigmentation in seed coat, hilum and pubescence (trichome hairs) is determined by three independent loci, *I* (*Inhibitor*), *R*, and *T* (*Tawny*). The *I* locus controls distribution of anthocyanin and proanthocyanidin pigments and comprises the multigenic, inverted repeat region of chalcone synthase (CHS) genes (*CHS1*, *CHS3* and *CHS4*) [1]. In its dominant form *I*, silences the expression of all nine CHS gene family via short interfering RNAs in a tissue-specific manner in the seed coat [2]. Since CHS is the first committed enzyme in the anthocyanin pathway, the *I* genotypes result in yellow seed coats. Colored soybeans have recessive *I* alleles and the various colors of the seed coats are influenced by the *R* and *T* loci. Black (*i,R,T*), imperfect-black (*i,R,t*), brown (*i,r,T*) and buff (*i,r,t*) seed coats. The *T* locus encodes a flavonoid 3′hydroxylase (*F3′H*) gene, the expression of which drives the synthesis of the anthocyanin cyanidin pathway branch [3].

Recently it has been proposed that *R* maps to a locus encoding a MYB transcription factor which may positively regulate the expression of a *UDP-glucose: flavonoid 3-O-glucosyltransferase* (*UF3GT*) gene that functions in the last step of anthocyanin synthesis [4]. These authors identified the recessive, brown *r* allele to affect the expression of a seed coat-specific R2R3 MYB transcription factor gene, Glyma09g36983 from the sequenced soybean genome as a strong candidate for the *R* locus. In addition, their qRT-PCR analysis supported a correlation between the mRNA levels of the putative *R* gene and a UDP-glucose: flavonoid 3-O-glucosyltransferase (*UF3GT*) gene (Glyma08g07130) that catalyzes the glycosylation of cyanidins to anthocyanins. The recessive *r* (brown) allele at Glyma09g36983 contained a single base deletion that would result in a truncated protein and did not upregulate this *UF3GT* gene. Based on a combined analysis of transcriptome and metabolome data, another recent study identified 20 anthocyanin, flavonoid and phenylpropanoid isogenes that were differentially expressed between black (*iRT*) and brown (*irT*) seeded soybean isolines [5]. This same study suggested that *R* locus candidate might be a transcription factor at the distal end of chromosome Gm09 at or near Glyma09g36840, 120 kb from the Glyma09g36983 MYB factor mapped by Gillman et al [4].

**Figure 1:**
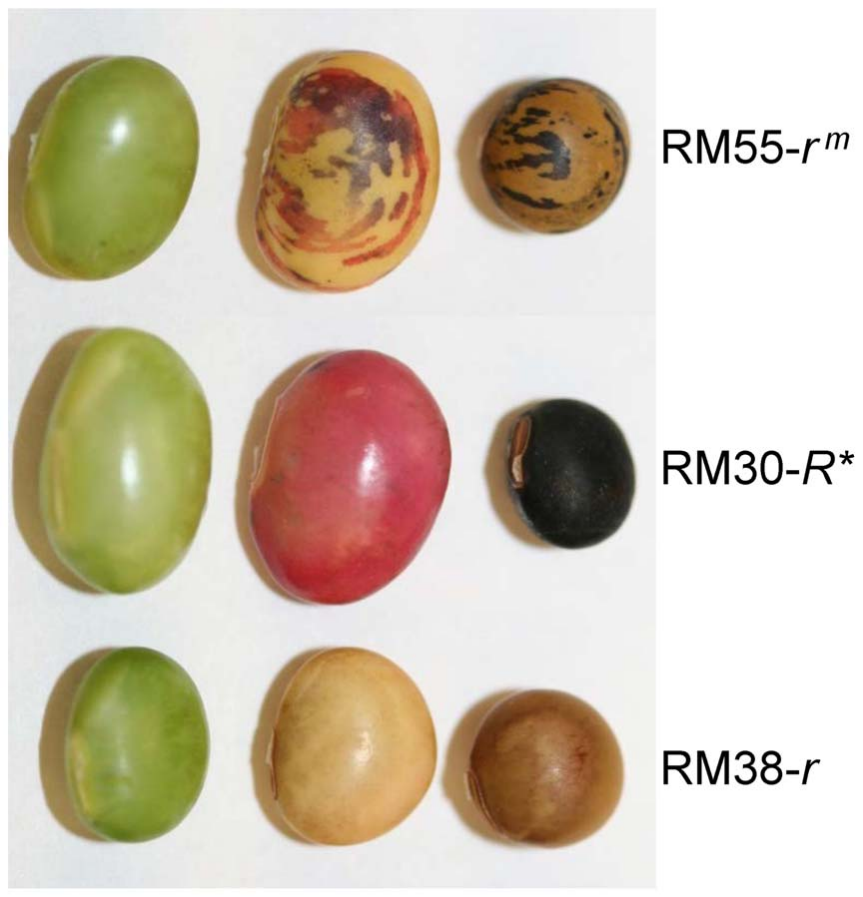
Phenotypes of Seeds Coats at Late Developmental Stages for Three Soybean Isolines. Differential anthocyanin expression in the seed coats of three soybean isolines with variants of the *R* locus alleles. RM55-*r^m^* with variegated black-brown seed coat; RM30-R* a stable revertant black-seeded isoline derived from RM55-*r^m^*; and RM38-*r* a brown seeded isoline used as the recurrent parent. See Table 1 for more information. Left, green seed of approximately 300–400 mg fresh weight; middle, dessicating seed of approximately 300–400 mg fresh weight; right, mature dry seed.

The *r^m^* allele of the *R* locus conditions a variegated or mutable distribution of black spots or concentric rings of black pigment superimposed on an otherwise brown seed coat (Figure 1). This phenotype is sometimes referred to as a striped phenotype. The *r^m^* allele present in a plant introduction line was backcrossed for five generations into a brown seeded Clark line with homozygous *i, r, T* genotype to create isoline L72-2040 with *i r^m^, T* genotype and released in 1972 by soybean breeder R.L. Bernard of the USDA Agricultural Research Service. We discovered that the *r^m^* variegated seed of L72-2040 demonstrated somatic and germinal instability to yield fully black seed [6]. One of these lines, here named as RM30, is shown in Figure 1 and the gene symbol R* is used to differentiate this stable revertant allele, derived from the *r^m^* line, from the standard *R* allele that specifics black seed coats. The mutable line is here referred to as RM55-*r^m^* and the brown isoline into which the *r^m^* allele was backcrossed is RM38-*r* (Figure 1).

Using primers based on the Glyma09g36983 as the *R* locus [4], we here report finding a large 13 kb CACTA transposable element insertion in the second intron of the MYB factor in the RM55-*r^m^* mutable soybean line along with a 929 bp PCR product representing excision products that restore the MYB transcription factor sequence. The transposon represents a second CACTA subfamily in soybean based on its transposase and subterminal repeat sequences that most resemble the first CACTA defective element described in soybean, *Tgm1* [7], and is more distant to *Tgmt* [8] and *Tgm9* [9]. Surprisingly the 13 kb CACTA transposon (designated TgmR*) is retained in the R* allele of a stable black-seeded revertant isoline. Using quantitative RNA-Seq data from a developmental series of seed coats from the black-seeded RM30-R* revertant and the RM38-*r* brown-seeded isoline, we also demonstrate that the MYB factor encoded by the *R* gene appears to induce the expression of anthocyanidin synthase (*ANS*) genes at late stages of seed development in the black RM30-R* line. Thus, our data further support Glyma09g3698 as the *R* locus in soybean and show that it regulates *ANS* genes and potentially other genes in the anthocyanin pathway despite a very low level of *R* gene transcripts. The *R* encoded MYB protein possesses four of the amino acids present in the subgroup five of MYB proteins that are involved in anthocyanin regulation by transcriptional activation [10].

Further we show that intron splicing was accurate despite the presence of the 13kb TgmR* element in Intron2 of the MYB gene. As determined by whole genome methylation sequencing, we show that the TgmR* sequence was relatively more methylated in the RM30-R* line than it is in the mutable RM55-*r^m^* progenitor line. Following mechanisms elucidated for the *Spm*-suppressible alleles and change of state alleles in maize [11], [12], [13], our data suggest that a transposase expressed from an element in the hypomethylated mutable RM55-*r^m^* line binds to the subterminal repeats and prevents intron splicing of the *R* gene (resulting in the brown seed coat background color) while it promotes excision events in some cells resulting in restoration of the MYB gene structure without the TgmR* element and expression of the functional MYB protein leading to black sectors and stripes. The stabilized RM30-R* revertant line, which had a higher level of methylation, either lacks the active transpose or it cannot bind to its subterminal repeats, thus allowing intron processing to proceed normally, although at a reduced level, resulting in enough of the MYB protein to stimulate anthocyanin production and thus black seed coats. In this regard, the TgmR* element that causes the variegated black and brown seed coats in soybean resembles McClintock's genetically and molecularly characterized *Spm-*suppressible and change of state alleles [reviewed in 12,13].

## Results

### The Mutable *r^m^* and Revertant R* Alleles Both Contain a Large Insertion in a MYB Transcription Factor Encoded by Glyma09g36983

**Figure 2:**
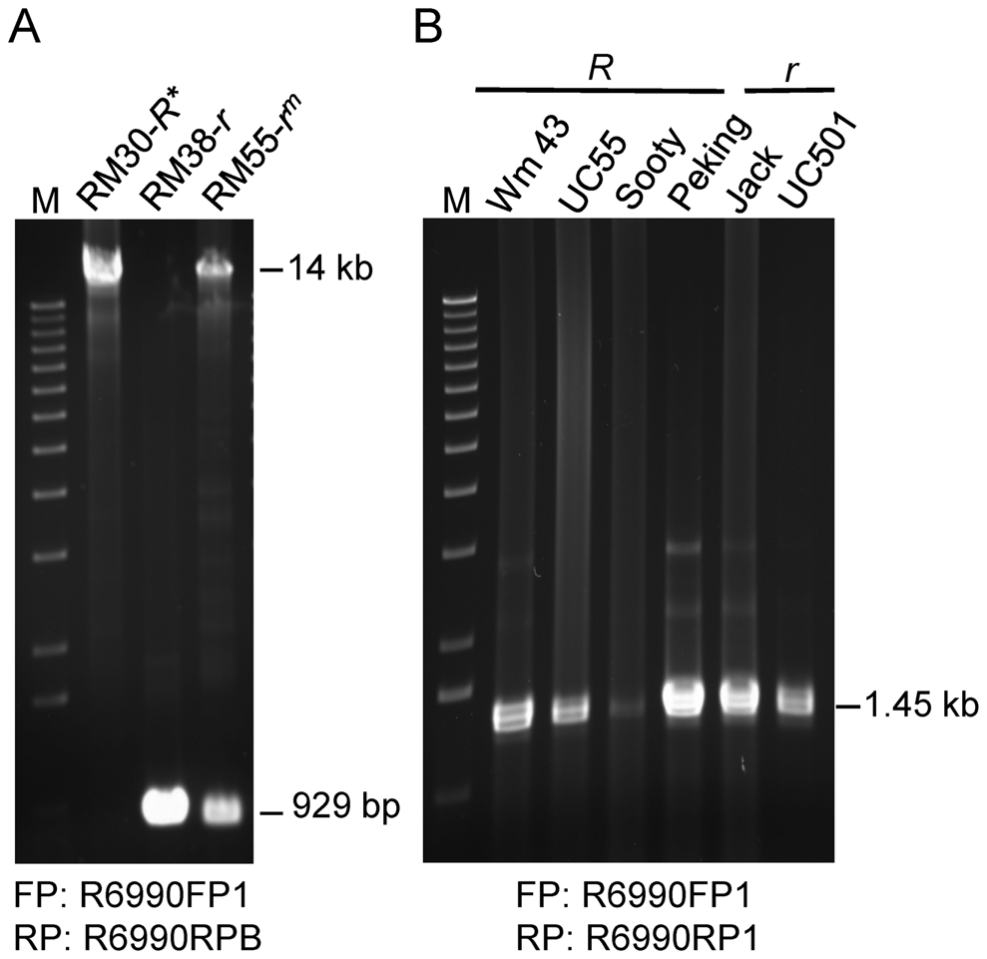
Amplification of a Large Insertion in the Glyma09g36983 Gene of RM55-*r^m^* and the Revertant Black RM30-R* Isolines. (A) Agarose gel displaying a ∼14 kb PCR fragment in the RM55-*r^m^* mutable and RM30-R* revertant alleles of the *R* locus. A small 929 bp fragment was the only amplification product from the *r* allele of the RM38-*r* brown-seeded line and it was the expected size of the DNA fragment comprised between the two oligo DNA primers (FP, forward primer: R6990FP1 and RP, reverse primer: R6990RPB) designed to amplify the 5′end portion of the Glyma09g36983 gene encoding a putative R2R3 MYB transcription factor. The 929 bp fragment was also an amplification product from the DNA of the mutable RM55-*r^m^*. These results predict an insertion of 13 kb in one allele of the mutable RM55-*r^m^* line that is maintained in the revertant RM30-R* black seed isoline. (B) Agarose gel displaying a 1.45 kb PCR fragment of the Glyma09g36983 gene amplified from four other soybean varieties with the standard *R* allele as well as from two soybean lines with an *r* allele (see Table 1 for full genotypes). The reverse primer in these instances was R6990RP1, situated 491 bp downstream from R6990RPB used in (A). The size marker “M” is a 1 kb ladder.

Figure 1 illustrates the phenotypes of the lines homozygous for the variegated *r^m^* allele (here designated as line RM55-*r^m^*) that was repetitively backcrossed into the genetic background of a brown seeded Clark isoline (RM38-*r*) and of a stable revertant R* allele (RM30-R*). Based on the report by Gillman et al. (2011) [4] that Glyma09g36983 (Gm09: 42562649–42564660 (+ strand) of the Williams 82 genome) is a potential candidate for the *R* locus, we designed primers to amplify this region from these three lines. Successful amplification was achieved with two long PCR primers (R6990FP1 and R6990RPB) and a Long Amplification (LA) PCR method [8] (see Methods and File S1). Figure 2A shows the two larger ∼14 kb DNA fragments amplified from RM30-R* and RM55-*r^m^* and the smaller 929 bp fragments in RM38-*r* and RM55-*r^m^*. The 929 bp fragments are the expected size of the DNA sequence between the two oligo DNA primers (FP:R6990FP1and RP:R6990RPB). Thus, the larger band revealed the existence of a DNA insertion in the RM30-R* and RM55-*r^m^* isolines somewhere in the Glyma09g36983 gene located between the 5′end and the first part of Intron2. The amplification was repeated with independent plant material to confirm the large insertion in the RM30-R* revertant line since the presence of a large insertion in the phenotype that mimics the wild type phenotype was unusual. The unadapted plant introduction source line for the *r^m^* allele (PI91073) was also checked (data not shown) and produced similar sized amplicons of 14 kb and 929 bp as found in the Clark line RM55-*r^m^* that is shown in Figure 2.

To examine whether such an insertion or any other macromolecular changes were present in other soybean lines carrying the alleles for black (*R*) or brown (*r*) pigments (Table 1), we amplified a large portion of the Glyma09g36983 in PCR reactions using the 37-nt forward primer R6990FP1 and a 31-nt reverse primer R6990RP1 located 491-nt downstream of the 37-nt R6990RPB primer (File S1). The expected PCR amplification product in the absence of insertions or deletions should be 1.45 kb in size. Figure 2B shows that all PCR amplified DNA fragments were of that size. Thus, the large insert seems to be specific for the *r^m^* allele or the RM30-R* line derived from it.

**Table 1:** Genotypes and phenotypes of soybean cultivars and mutant isolines used in this study.

Lab name	Genotype	Seed Coat Phenotype	Cultivar/origin PI number	PI number
RM55	*i,r^m^,T*	Black-brown striped	Clark isoline L72-2040	PI547559
RM30	i,R*,T	Black	Revertant in L72-2040	NA
RM38	*i,r,T*	Brown	Clark isoline L67-3484	PI547494
PI91073	*i,r^m^,T*	Black-brown striped	Source of *r^m^*allele	PI91073
Wm43	*i^i^,R,T*	Black hilum	Williams	PI548631
UC55	*i,R,T*	Black	Mutation in Williams	PI518670
Sooty	*i,R,T*	Black	Sooty	PI548415
Peking	*i,R,T*	Black	Peking	PI417243
Jack	*I,r,T*	Yellow	Jack	PI540556
UC501	*I,r,T*	Yellow	Harosoy	PI548575
T157	*i,R,t*	Imperfect black	Mutation in Richland	PI548182

All cultivars are homozygous for the alleles indicated. The varieties are searchable by the PI number in the USDA Germplasm Resources Information Network (GRIN). NA, not applicable.

L numbers are isoline number designations. The *r^m^* allele from PI91073 that specifies a variegated phenotype had been repetitively backcrossed into the Clark brown seeded isoline L67-3484 (here designated as RM38 with the *r* allele) to create the variegated Clark isoline L72-2040 (here designated as RM55 with the *r^m^*) by R.L. Bernard of the USDA. The L72-2040 line demonstrated somatic and germinal instability to yield fully black seed. One of these lines is here designated as RM30 and the gene symbol R* is used to designated this revertants of the *r^m^* line from the standard *R* allele that specifics black seed coats.

### A 13 kb Insertion in the R* and *r^m^* Alleles is a Novel Transposon of the *Tgm1* Subfamily of CACTA Elements

The ∼14 kb and 929 bp DNA fragments amplified from the mutable line RM55-*r^m^*, the stable revertant isoline RM30-R*, and the RM38-*r* self-brown soybean line (Figure 2A), were extracted from the gel pieces and cleaned using the Zymoclean Large Fragment DNA Recovery Kit protocol. The two larger ∼14 kb DNA fragments were sequenced using the 454 method at the W. M. Keck Center for Comparative and Functional Genomics at the University of Illinois at Urbana-Champaign (see Methods section and Table S1). The 929 bp PCR fragments were sequenced using the Sanger method at the same sequencing facility. Contigs assembled from sequence reads of whole genome resequencing (Table S1) also allowed confirmation of the structure of these amplicons from RM30, RM55 and RM38.

**Figure 3:**
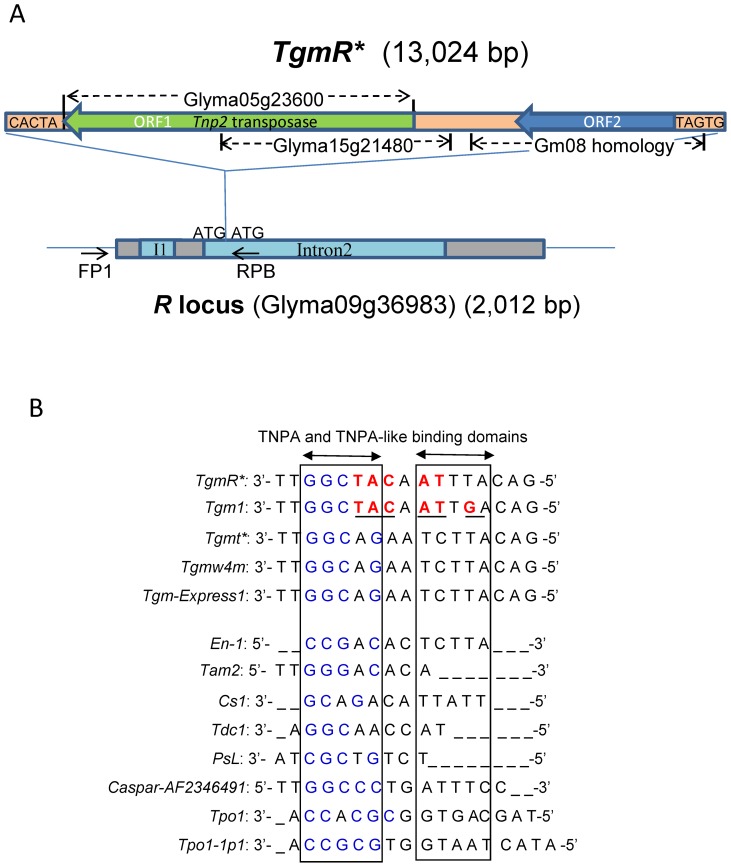
The 13 kb CACTA Insertion, Designated TgmR*, and its Subterminal Repeats Within the *r^m^* and R* Alleles. (A) Schematics of the CACTA transposon (TgmR*) insertion in Intron2 of the *R* locus encoding a R2R3 MYB transcription factor. The *R* locus gene, Glyma09g36983, depicted in gray (exons) and blue (introns) has a 13 kb CACTA transposon insertion 110 bp from the beginning of Intron2 in the *R* locus of both the variegated RM55-*r^m^* and the black-seeded revertant RM30-R* soybean isolines. The ATG duplication is shown at the site of insertion. The locations of the two primers used to amplify the 14 kb fragment containing the transposon insertion are depicted by the two small arrows, FP1 at the 5′ UTR and RPB in Intron2 near the insertion site. The 13 kb TgmR* transposon is a CACTA element based on the terminal ends sequences and the *Tnp2* transposase it encodes. Both the location of the transposase and the end sequences are marked. Sequences of high homology to this transposase region were found in chromosomes Gm05 and Gm15 and the Glyma models predicted for the Williams 82 genome are indicated. Likewise, the 3′ end of TgmR* has high homology to a sequence in chromosome Gm08 and to *Tgm1*, another smaller CACTA element of the soybean. Green and blue arrows represent the putative ORF1 (Tnp2 transposase) and ORF2 respectively. (B) Alignment of multiple CACTA transposon subterminal repeated motifs. The consensus sequences of the TgmR* subterminal direct repeats were aligned to other CACTA consensus sequences from other *Glycine max* transposons (Tgm1, Tgmt*, Tgmw4m and *Tgm-Express1*) in addition to other subterminal repeats from CACTA transposons in other plants. Portions of the sequence domains for TNPA-like protein binding are boxed. One of these motifs are GC rich (left) and the second AT rich (right). Based on the similarities of these terminal repeat motifs, it is clear that TgmR* is more closely related to *Tgm1* than to the other three characterized *G. max* transposons and all of the other CACTA transposon motifs in the lower panel.

The contig assembly resulting from sequencing the ∼14 kb fragment in the RM30-R* isoline with black seeds revealed the insertion of a 13 kb CACTA transposon near the beginning of Glyma09g36983 Intron2 (File S1) as illustrated in Figure 3A. This newly identified CACTA transposon that we named TgmR* is 13,024 bp in length (File S2) and has the characteristic CACTA ends and a three-base-target site duplication (ATG). In addition, it features reiterated direct and inverted sequence motif in the subterminal regions able to form secondary DNA/RNA stem-loop structures (Files S3).

The consensus sequence of the motif in the repeats is shown at top of Figure 3B where it is compared to the consensus sequence motifs of other CACTA transponsons studied in *G. max* and other plant species. In a previous study of an autonomous 20.5-kb CACTA element (Tgmt*) in *G. max* [8], we noticed a clear distinction in the consensus sequence motif between three characterized CACTA transposons (Tgmt*, *Tgmw4m* and *Tgm-Express1*) and *Tgm1*. The *Tgm1* sequence motif diverged from all three others in six nucleotide positions shown in red type in Figure 3B. This suggested the existence of two distinct CACTA transposon families in soybean. The TgmR* sequence motif is almost identical to the one in *Tgm1* [7], [14] with five of the six divergent nucleotides (nt) being identical in both, TgmR* and *Tgm1*, implying that they are more closely related and belong to the same subfamily of *G. max* CACTA elements (Figure 3B). Further evidence is the more than 1,000-nt of sequence identity between TgmR* and *Tgm1* at the 3′end (File S2) and more than 100 continuous-nt at the 5′end of the transposon. The remainder of the *Tgm1* DNA sequence, a defective element of 3,556-nt total size, deviates considerably from the TgmR* sequence.

To determine with more accuracy the total coding capacity of this 13 kb newly identified CACTA transposon TgmR*, we used the Softberry (http://linux1.softberry.com/berry.phtml) gene prediction program to find two putative ORFs. A large ORF1 with 10 or 11 exons (from nt 920 to 7,481) and a small ORF2 with three exons (from nt 9,347 to 12,255). The larger ORF1 predicted using *Glycine max*, *Arabidopsis* and *Vitis vinifera* genomes as models, coincides with the predicted transposase of the tnp2 family. We previously identified another potentially autonomous CACTA transposon, Tgmt*, inserted in an *F3′H* (flavonoid 3′ hydroxylase) allele to be 20.5 kb in size and encoding a transposase with tnp2 and TNP1/EN/SPM domains [8] (Acc. No. EU190440). This same transposon was found inserted at the *W4* locus encoding dihydroflavonol-4-reductase 2 (DFR2) and was called *Tgm9* [9]. However, DNA sequence alignment between Tgmt* and TgmR* showed little similarity between the two elements except for the transposase sequence region where a stretch of 3,040 bp (Tgmt*: 3373….6413; TgmR*: 3299….6348) was 71% similar at the nucleotide level. This divergence is additional evidence in support of two distinct CACTA transposon families in soybean.

To determine the redundancy of the TgmR* sequence in the Williams 82 genome we attempted a BLAST search in Phytozome [15] using its “unmasked” feature to allow repetitive DNA alignments. The sequence, or part of it, is so repeated that no result was returned when the entire TgmR* sequence was used. However, when the BLAST search was done with portions of the TgmR* sequence, then, many repeats all through the Williams 82 chromosomes showed sequence similarity. The most extensive regions of sequence similarity to TgmR* in the Williams 82 genome were found in chromosomes Gm05 and Gm15, where Glyma05g23600 (6,412 nt) and Glyma15g21480 (5,909 nt) reside, respectively. Glyma05g23600 is annotated as related to transposase family tnp2 and 6,406 nt of its sequence are comprised in TgmR* (Figure 3A). Glyma15g21480 has the same annotation but its sequence lacks 2,886-nt of the Glyma05g23600 3′-end sequence. The TgmR* sequence similarity to Glyma15g21480 extends 668-nt upstream of the Glyma05g23600 5′-end sequence similarity (Figure 3A). In addition, the TgmR* right border has similarity to a 4,191 nt sequence fragment in Gm08 (94002..98330) with no predicted gene annotation (Figure 3A).

Another previously characterized *G. max* deletion derivative transposon, *Tgm5* that is 1,002-nt in size [16] (Acc.No. X13528.1), also contains an entire tnp2 transposase domain that aligns with 89% similarity to a 1,002-nt region of TgmR* (5,952–6,954-nt) and Glyma05g23600 (Gm05:29,250,569..29,251,571) but with 93% similarity to a 1,002-nt region of Glyma15g21480 (Gm15:19,6 88,859..19689861). Both these genes in Gm05 and Gm15 have the tnp2 transposase highly similar (89 or 93%) to the *Tgm5* tnp2 transposase domain. It was estimated [16] that *Tgm5* is 39% similar to the ORF1 of the *Zea mays En-1/Spm* transposable element which is the first autonomous CACTA element in maize and corresponds to one of McClintock's original transposable element systems. In addition to those two loci in Gm05 and Gm15, the 1,002-nt sequence portion (*Tgm5*) with the tnp2 transposase domain is repeated with some variability 81 times in Gm05 and 93 times in Gm15. In a similar fashion, that same sequence is repeated in all other 18 chromosomes of the *G. max* Williams 82 genome.

The results of sequencing the 929 bp amplification fragments derived from either the RM55-*r^m^* mutable line or the RM38-*r* line uncovered a significant difference between the two sequences, mainly a “C”-nt deletion in Exon2 of the RM38-*r* line with the brown seed color phenotype. This base deletion causes a frame shift resulting in a prematurely terminated translation product of only 85 amino acids, 152 amino acids shorter than the mature, intact protein with 237 amino acids. In contrast, the 929 bp fragment amplified from the RM55-*r^m^* mutable black and brown striped seeded line has the “C”-nt that is deleted in the RM38-*r* line revealing a divergence between the genetic sources of those two soybean lines. The RM38-*r* line (self-brown L67-3484) line that was used to introgress the mutable (*r^m^*) allele has the same “C”-nt deletion in Exon2 as described by Gillman et al (2011) in mapping the *R* locus alleles.

### Differential Expression of the MYB Transcription Factor in Immature Seed Coats of the Black Revertant (RM30-R*) and Brown (RM38-*r*) Isolines

**Figure 4:**
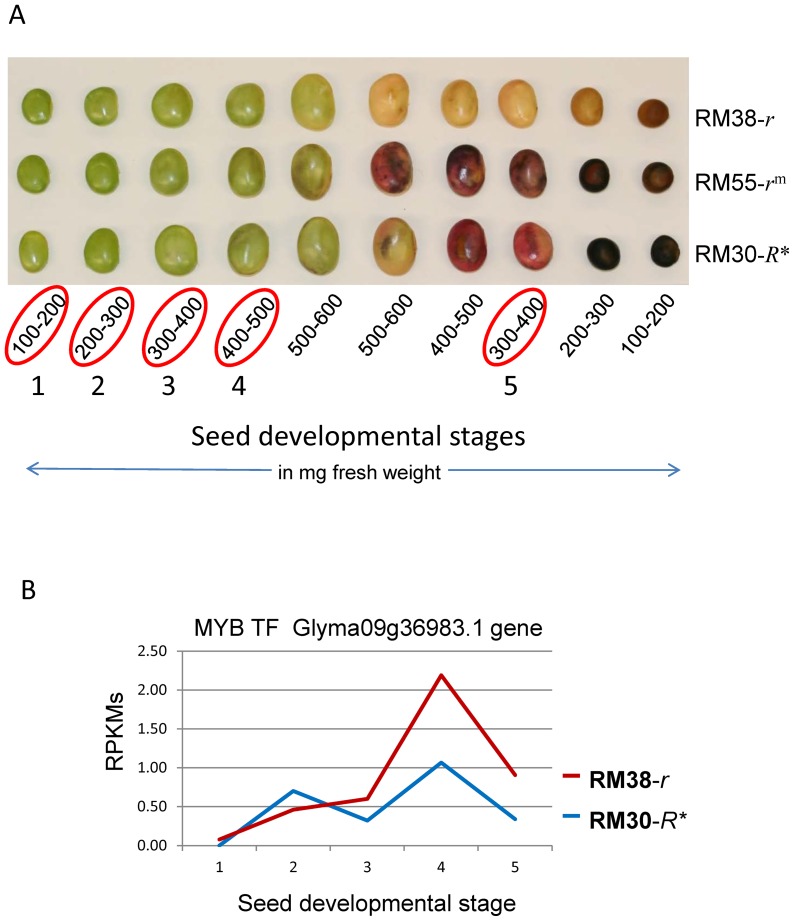
Differential Expression of the MYB Transcription Factor Encoded by the *R* Locus in Developing Seed Coats of a Stable Black-Seeded Soybean Revertant Line RM30-R* and a Brown-Seeded Line RM38-*r*. (A) Soybean seed developmental stages for the RM38-*r* (brown), RM55-*r^m^* (variegated) and RM30-R* stable black revertant. Encircled in red are the five developmental stages (in mg seed fresh weight) chosen for the RNA-Seq analysis. See Table S1 for the sequence read counts of each sample which ranged from from 30 to 77 million. (B) Expression of Glyma09g36983 in RPKMs plotted against the five stages of seed development in mg seed fresh weight as shown in (A) above. 1: 100–200 mg, 2: 200–300 mg, 3: 300–400 mg, 4: 400–500 mg and 5: 300–400 mg as the seeds enter desiccation. The solid red line represents transcripts derived from seed coats of the RM38-*r* brown-seeded line without the TgmR* but with a “C”-nt deletion in Exon2. The blue line represent the expression in seed coats of the RM30-R* black revertant line with the TgmR* insertion in Intron2.

We compared the expression Glyma09g36983 MYB transcription factor in the seed coats of the black revertant RM30-R* soybean line to that in the defective brown RM38-*r* soybean line at various stages of seed development. Total RNA was extracted from seed coats at five stages of seed development (Figure 4A) following a modified method that prevents RNA adhesion to the proanthocyanidins (tannins) that accumulate in the vacuoles of pigmented seed coats [17], [18]. The five stages of seed development were chosen based on the fresh weight of entire seeds: 100–200, 200–300, 300–400 and 400–500 mg with green cotyledons, and a later stage in which seed desiccation had initiated judging by the yellowing of cotyledons and a lower fresh weight of 300–400 mg.

The RNA-Seq results reflecting the level of transcript reads aligning to the Glyma09g36983 (Glyma09g36990 in Phytozome *G. max*_109 v.1a-1.0) sequence are given in RPKMs (reads per kilobase of gene model per million mapped reads) as shown in in Table 2 (top row) and graphically in Figure 4B. This MYB transcription factor was not expressed at the early stages examined (100–200 mg) and at very low levels later in development when the anthocyanins and proanthocyanins start pigmenting the seed coat. The quantitative levels were 1.07 RPKM in the RM30-R* black revertant that contains the TgmR* insertion and only 2.19 in the RM38-*r* brown isoline. This RPKM ratio of 0.49 (p-value: 0.069) indicates no significant differences of expression of the Glyma09g36983 gene in the RM30-R* (black) and RM38-*r* (brown) seed coats at the peak of its measured expression in the 400–500 mg seed weight.

**Table 2:** Differential RNA expression of anthocyanin pathway genes in developing seed coats of lines RM30-R* (black) and RM38-*r* (brown).

	RM30-R* RPKMs in wt. range					RM38-*r* RPKMs in wt. range					RM30/RM38 p-value	Annotation		Locus
Gene Model	100	200	300	400	300	100	200	300	400	300	400					
Glyma09g36983.1	0.00	0.70	0.32	**1.07**	0.34	0.08	0.46	0.60	2.19	0.91	0.49		0.069		MYB	*R*
Glyma09g36966.1	0.00	0.16	0.06	0.10	0.00	0.03	0.10	0.00	0.40	0.15	0.25		0.384		MYB	
Glyma09g37010.1	0.00	0.00	0.00	0.00	0.00	0.00	0.00	0.00	0.00	0.11	0.00		1.000		MYB	
Glyma18g49670.2	0.00	0.00	0.00	0.00	0.00	0.00	0.00	0.00	0.00	0.11	0.00		1.000		MYB	
Glyma01g43880.1	509.11	212.43	210.65	122.96	19.46	526.37	120.74	41.10	44.44	20.76	2.77		7.59 E-11		CHS7	*I*
Glyma11g01350.1	382.08	135.35	138.73	75.69	13.92	395.90	76.34	25.61	28.47	14.87	2.66		6.78 E-10		CHS8	*I*
Glyma20g38560.1	5.43	13.65	11.07	17.57	11.87	7.58	17.15	9.33	12.24	7.73	1.43		0.927		CHI1A	
Glyma20g38580.1	14.65	46.66	23.13	36.87	10.31	21.70	39.93	11.63	12.26	9.30	3.01		1.75 E-10		CHI2	
Glyma06g21920.1	71.05	11.73	14.52	4.42	1.14	61.60	4.07	1.59	3.33	0.79	1.33		0.302		F3′H	*T*
Glyma13g04210.1	0.12	0.08	0.08	4.66	4.87	0.18	0.68	0.01	0.04	0.34	4.66		1.15 E-33		F3′5′H	*W1*
Glyma02g05450.1	326.86	162.99	119.24	124.64	20.30	340.30	90.65	26.45	38.72	14.52	3.21		6.24 E-14		F3H	*Wp*
Glyma14g07940.1	76.21	66.14	60.19	45.78	2.64	76.98	35.18	15.30	14.63	1.52	3.13		5.16 E-12		DFR1	*W3*
Glyma17g37060.1	4.71	1.19	0.33	3.92	2.87	3.56	1.65	0.06	0.35	0.15	3.92		4.98 E-14		DFR2	*W3*
Glyma01g42350.1	76.09	36.74	12.86	**35.41**	11.00	76.07	24.19	2.17	**3.48**	1.47	**10.17**		**2.12 E-38**		ANS2	
Glyma11g03010.1	88.80	42.81	17.37	**39.75**	9.61	91.77	26.93	2.07	**3.78**	1.03	**10.51**		**2.71 E-40**		ANS3	
Glyma08g07130.1	2.01	5.55	7.68	12.79	12.51	3.40	12.11	7.78	9.87	3.66	1.29		0.281		UGT78K2	
Glyma07g30180.1	1.43	0.67	1.39	0.97	1.04	1.93	1.45	1.04	0.94	0.32	1.03		0.915		UGT78K1	
Glyma08g06630.1	0.98	0.09	0.40	0.49	0.17	1.27	0.16	0.04	0.55	0.23	0.89		0.712		ANR1	
Glyma08g06640.1	0.06	0.00	0.02	0.04	0.00	0.06	0.00	0.00	0.03	0.00	1.33		0.972		ANR2	
Glyma05g36210.1	58.27	36.87	47.81	17.85	4.02	43.20	15.16	8.66	4.77	2.27	3.74		6.33 E-12		AOMT	

RPKMs are shown for seed coats from seed of the five seed fresh weight ranges denoted 100–200, 200–300, 300–400, 400–500 mg and 300–400 mg desiccating seed coats as shown in Figure 4A. Ratios and p-values are shown for the 400–500 mg weight range, and date for the ANS genes at this stage are in Bold.

Glyma09g36983 in Phytozome *G. max*_189 a1_1.1 was previously named Glyma09g36990 in Phytozome *G. max*_109 a1_1.0.

Glyma09g36966 in Phytozome *G. max*_189 a1_1.1 was previously named Glyma09g36970 in Phytozome *G. max*_109 a1_1.0.

A biological replicate of the 400–500 mg weight range also showed very low levels in each genotype, although the differential expression was greater with 0.38 RPKM in the RM30-R* black revertant line versus 3.7 in the RM30-*r* brown isoline (Table 3). Thus, the *r* allele of the brown seed coats appears to be expressed at a higher level than the R* allele that is interrupted by the TgmR* element, although phenotypically, the RM30-R* black revertant line more closely resembles black seed coats with the standard *R* allele. Table 3 also shows the results of RNA-Seq for two soybean lines containing the standard *R* allele with black seed coats, T157 and UC55. These lines have RPKMs ranging from 2.55 to 5.9 in two of the surveyed weight ranges near the peak of *R* gene expression. Thus, this MYB factor has relatively low expression even in the fully functional standard *R* alleles with black seed coats. The lower level of *R* gene expression in RM30-R* could be explained by the TgmR* insertion in Intron2 reducing functional transcript levels from the allele. However, the lower level is still sufficient to produce black seed coats.

**Table 3:** Differential RNA expression of Glyma09g36983 and related MYB transcription factor genes in seed color isolines.

Cultivar	RM30-R*				RM38-*r*				T157-*iR*		UC55-*iR*	
Phenotype	black				brown				imp. black		black	
Weight range	300^1^	300^2^	400^1^	400^2^	300^1^	300^2^	400^1^	400^2^	300	400	300	400
Glyma09g36983.1	0.32	0.12	1.07	0.38	0.60	1.91	2.19	3.70	3.26	2.55	2.73	5.90
Glyma09g36966.1	0.06	0.04	0.1	0.04	0	0.47	0.4	0.93	0.48	0.17	0.21	0.71
Glyma09g37010.1	0	0	0	0	0	0.02	0	0	0	0	0	0
Glyma18g49670.1	0	0	0	0	0	0	0	0	0	0	0	0

RPKMs are shown for seed coats from seed of two seed fresh weight ranges denoted 300–400 and 400–500 mg as shown in Figure 4A.

Superscripts 1 and 2 for the 400–500 mg seed fresh weight represent two independent RNAseq extractions and sequencing determinations.

Glyma09g36966, Glyma09g37010, and Glyma18g49670 have transcript sequence similarities of 90%, 87%, and 84%, respectively, to Glyma09g36983.

Glyma09g36983 in Phytozome *G. max*_189 a1_1.1 was previously named Glyma09g36990 in Phytozome *G. max*_109 a1_1.0.

Glyma09g36966 in Phytozome *G. max*_189 a1_1.1 was previously named Glyma09g36970 in Phytozome *G. max*_109 a1_1.0.

We examined the expression of three MYB transcription factors, Glyma09g36966, Glyma09g37010, and Glyma18g49670 that have transcript sequence similarity of 90%, 87%, and 84% respectively to Glyma09g36983. The level of expression of these three genes as shown in Tables 2 and 3 is even lower or nil, and the RPKM values associated with Glyma09g36966 may reflect the number of sequence reads aligning to the 530-nt stretch with 90% transcript sequence similarity to Glyma09g36983. Three mismatches was the cutoff used to determine the number of RNA sequence reads aligning to a given gene transcript using Bowtie. A more stringent alignment search with zero mismatches supported the above statement and strongly suggested that none of the three related MYB transcription factors are expressed in the soybean seed coats.

### Differential RNA Expression of Key Genes in the Anthocyanin and Proanthocyanidin Metabolic Pathways between the Black (RM30-R*) and Brown (RM38-*r*) Seed Coats

Once we had evidence that the TgmR* insertion slightly suppressed the expression of the *R* locus gene, Glyma09g36983, at late stages of seed development, we questioned at which point in the anthocyanin biosynthetic pathway the encoded MYB transcription factor exerted its regulatory control. Towards that end, we compared the level of mRNA expression of well characterized genes in the pathway in the RNAseq data sets obtained from the black and brown seed coats of the RM30-R* revertant and RM38-*r* defective line, at all five stages of seed development under study (Table 2).

**Figure 5:**
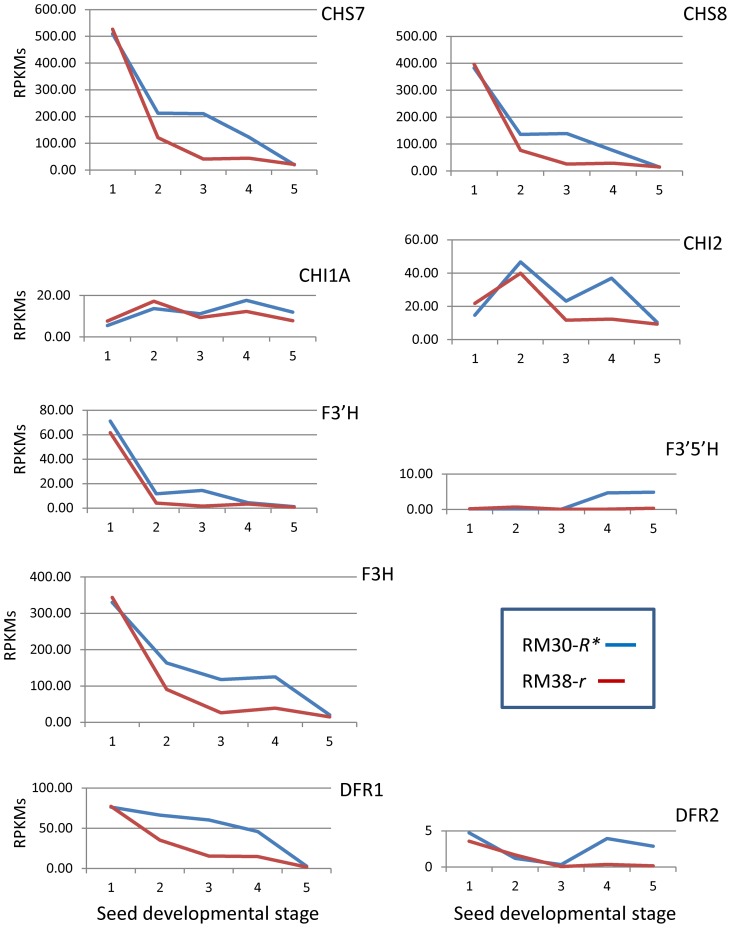
Differential Expression of Genes that Function in the Early Steps of the Anthocyanin and Proanthocyanidin Biosynthetic Pathways Between the Black-Seeded RM30-R* and Brown-Seeded RM38-*r* Lines. Transcript levels are in RPKMs plotted against the same five stages of seed coat development as shown in Figure 4 for RM30-R* where the R* allele is interrupted by the 13 kb TgmR* insertion (profile in blue) and the RM38-*r* line where the *r* allele is not interrupted by TgmR* but has a “C”-nt deletion in Exon2 (red profile). Graphs have different scales. The Glyma models for the indicated genes are as follows: *CHS7* (Glyma01g43880.1); *CHS8* (Glyma11g01350.1); *CHI1A* (Glyma20g38560.1); *CHI2* (Glyma20g38580.1); *F3′H* (Glyma06g21920.1); *F3′5′H* (Glyma13g04210.1); *F3H* (Glyma02g05450.1); *DFR1* (Glyma14g07940.1); *DFR2* (Glyma17g37060.1. See Figure 7 for the pathway abbreviations.

**Figure 6:**
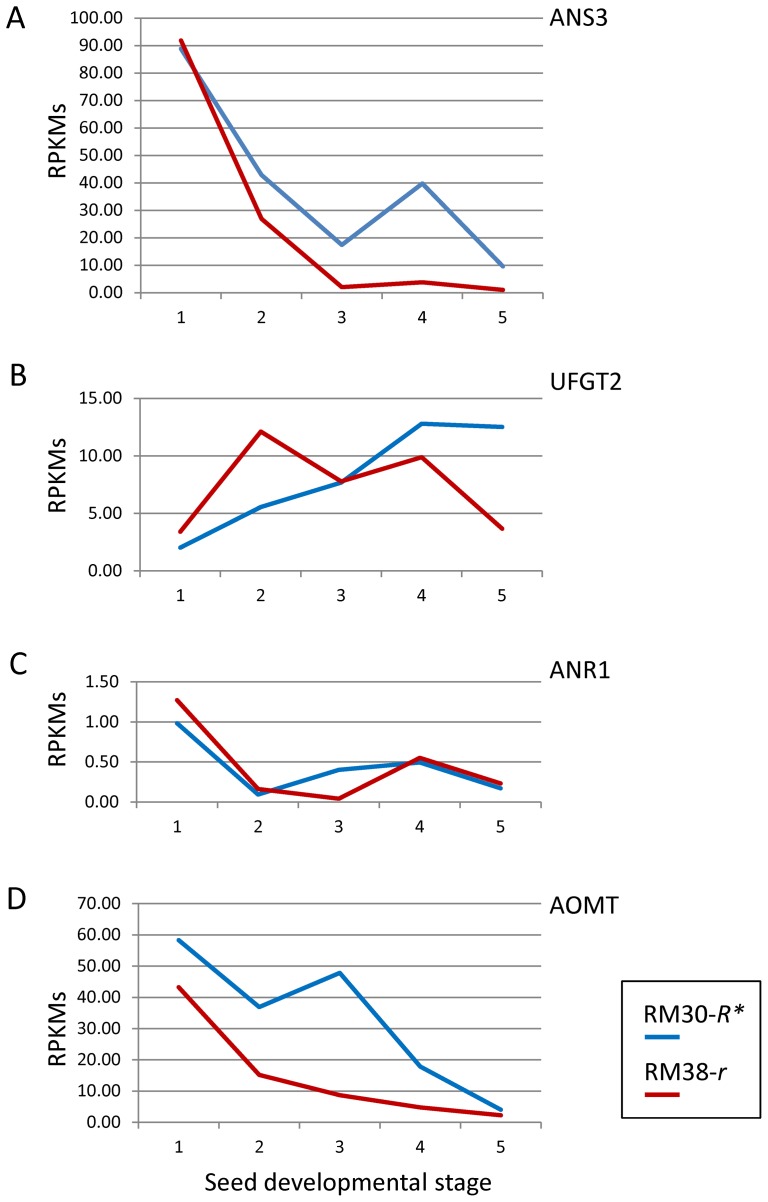
Differential Expression of Four Genes That Function in the Last Steps of Anthocyanin and Proanthocyanidin Synthesis Between the Black Seeded RM30-R* and Brown Seeded RM38-R* Lines. Transcript levels are in RPKMs plotted against the same five stages of seed coat development as shown in Figure 4 for RM30-R* where the R* allele is interrupted by the 13 kb TgmR* insertion (profile in blue) and the RM38-*r* line where the *r* allele is not interrupted by TgmR* but has a “C”-nt deletion in Exon2 (red profile). Graphs have different scales. The Glyma models for the indicated genes are as following. (A) ANS3 (Glyma11g03010.1); (B) UFGT2 (Glyma08g07130.1); (C) ANR1 (Glyma08g06630.1) and (D) AOMT (Glyma05g36210.1. See Figure 7 for the pathway abbreviations.

**Figure 7:**
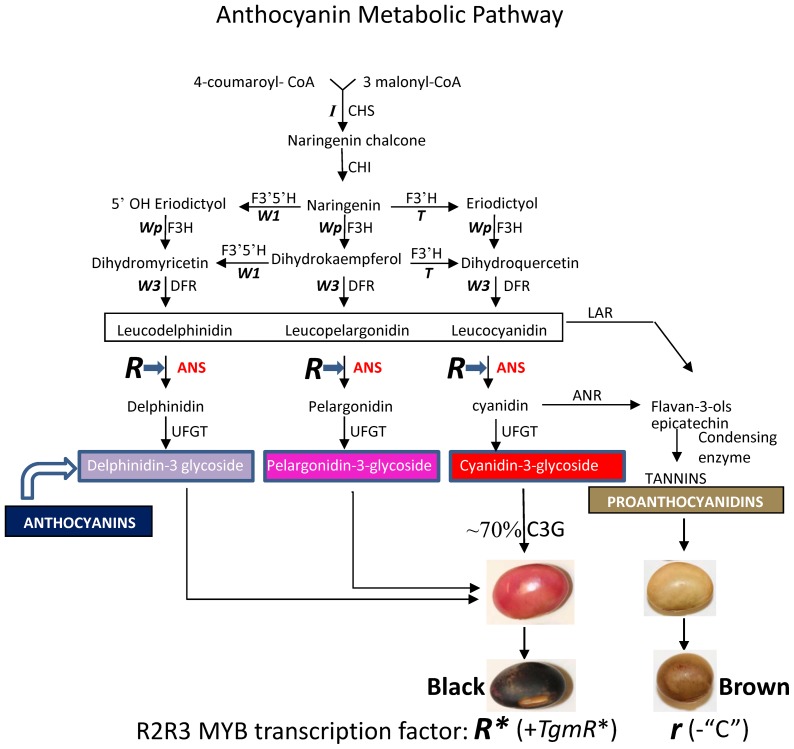
Diagram of the Soybean Seed Coat Anthocyanin Metabolic Pathways and Enzymatic Steps Regulated by the MYB Transcription Factor Encoded by the R* Allele in the Black Seeded Line RM30-R*. In display are the three branches leading to the synthesis of the purple delphinidins, pink pelargonidins, red cyanidins as well as the brown proanthocyanidins. The major anthocyanin in black soybean seed coats is cyanidin-3-glycoside (C3G) at ∼70%. The brown seed coats contain mostly proanthocyanidins. Enzymes and seed coat color loci are abbreviated in uppercase letters: *I*, chalcone synthase (CHS); *W1*, flavonoid 3′5′-hydroxylase (F3′5′H); *T*, flavonoid 3′-hydroxylase (F3′H); *Wp*, flavanone 3-hydroxylase (F3H); *W3*, dihydroflavonol-4-reductase (DFR); Annthocyanidin synthase (ANS) also called leucoanthocyanidin dioxygenase (LDOX); UDP-flavonoid glucosyltransferase (UFGT); Leucoanthocyanidin reductase (LAR); Anthocyanidin reductase (ANR). The MYB transcription factor encoded by the R* allele Glyma09g36983 gene enhances expression/accumulation of ANS transcripts as shown in Figure 6.

Contrary to the difference observed for the expression of the MYB gene in the two lines in Figure 4B, where higher levels were measured in the brown RM38-*r* seed coats, all other pathway genes analyzed had higher RPKM expression values in the black RM30-R* line at the late stages of seed development (Figures 5 and 6, Table 2). These increases occurred at the developmental time when the *R* locus gene, Glyma09g36983, is activated. One exception is the *ANR* genes for which the low level of expression at the late stages of seed development was maintained in the seed coats of the black RM30-R* line as well as in the brown RM38-*r* line (Table 2). This is consistent with the function of this gene's product in directing the synthesis of proanthocyanidins now in competition for its substrate that is being diverted towards anthocyanin synthesis in the black seed coats at the late stages of seed development as shown in Figure 7.

Several of the genes involved in the anthocyanin pathway (*CHS*, *F3′H*, *F3H*, *DFR*, *ANS*, *ANR1* and *AOMT*) are expressed at higher level early in seed development (100–200 mg seed fresh weight) and the amount of transcripts decline in seed coats of both lines as seed growth advances (Figures 5 and 6). However, this decline is arrested or reversed for all these genes in the seed coats of the black-seeded RM30-R* line at the third and fourth stages of seed development. For those genes that are not expressed or expressed at very low level earlier in development (*CHI*, *F3′5′H* and *UFGT*), the expression increased to a slightly higher level in the seed coats of the black RM30-R* seeds during the late stages of seed development. Despite the two *UFGT* genes (*UGT78K2* and *UGT78K1*) transcript sequences being 92% similar, the sequence reads from the seed coat RNA samples aligned better to the *UGT78K2* transcript and differentially between the two lines (Table 2 and Figure 6). This suggests that the *UGT78K1* gene may not be expressed in the seed coat but rather in the cotyledons as it has been reported previously [4].

Although all the mentioned genes appear to be differentially expressed in the seed coats of the black and brown seeds, the ones that show the highest RPKM ratios and lowest p-values are the *ANS* genes with an RM30/RM38 of 10.17 (p-value 2.12 E-38) and 10.51 (p-value 2.71 E-40) respectively, at the 400–500 mg seed weight (Table 2). The transcript sequences of the two *ANS* genes are 94% similar and it is not surprising that the numbers of RNA sequence reads for each gene (*ANS2* and *ANS3*) are very similar and show parallel patterns of expression in the five samples measured from the two lines (RM30-R* and RM38-*r*). Figure 6 shows the differences in the number of sequence reads in RPKMs for the *ANS3* gene in the RM30-R* black and the RM38-*r* brown seed coats.

### Molecular Domains in the *R* Locus-Encoded MYB Transcription Factor are Predictors of its Regulatory Role in Anthocyanin Synthesis

**Figure 8:**
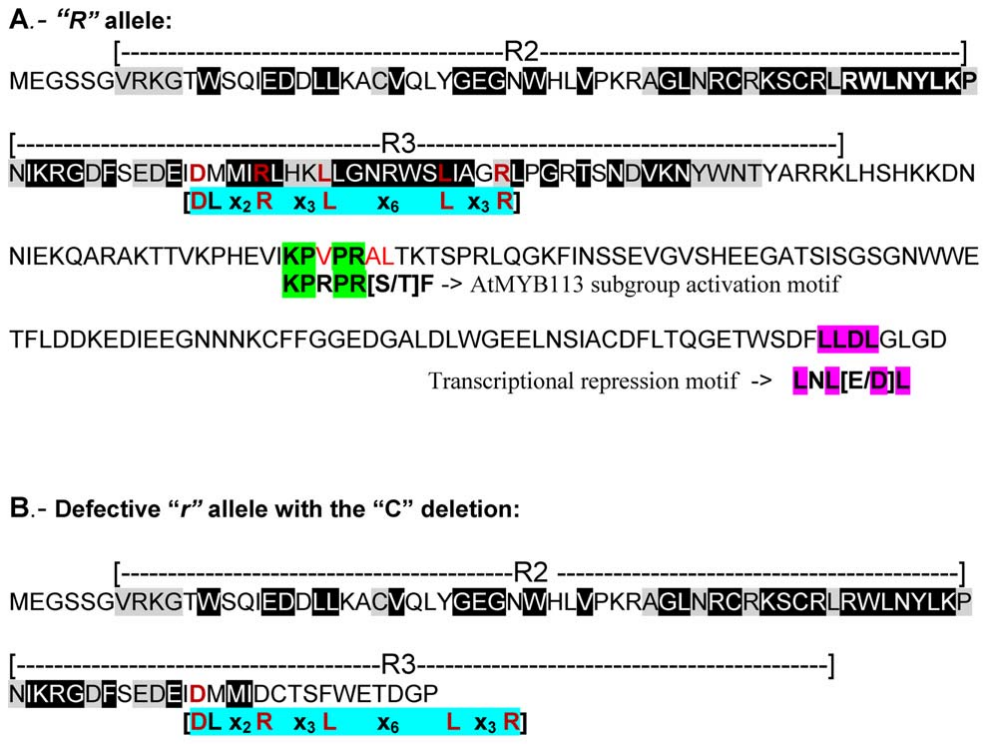
Features of the R2R3-MYB Transcription Factor Protein Sequence Encoded by the *R* Locus, Glyma09g36983. (A) The full length standard *R* allele. The extent of the imperfect R2 and R3 repeats is marked with the two dotted lines. Highlighted in black and gray are the conserved amino acids found in other higher plant MYB TFs of the R2R3 class (Lin-Wang et al, 2010). The amino acids highlighted black are best conserved. Aqua highlight is the [D/E]Lx_2_[R/K]x_3_Lx_6_Lx_3_R conserved amino acid signature functionally relevant in the MYB TF interaction with R/B-like bHLH proteins [23]. The conserved amino acids of this signature in the *R* locus encoded MYB are indicated in burgundy type. Green highlights the amino acids that are part of the motif found in AtMYB113 of subgroup 5 involved in anthocyanin regulation (activation) [10]. The divergent amino acids are indicated in red type. Highlighted fuchsia are four of the five amino acids constituting the transcriptional repression motif found in AtMYBs of subgroup 4 (AtMYB3, AtMYB4, AtMYB7, AtMYB32) [10]. (B) The truncated polypeptide resulting from the Exon-2 “C”-nt deletion in the defective *r* allele. It preserves only the R2 domain and 16 amino acids of the R3 domain.

We searched for molecular domains and motifs in the *R*-encoded MYB protein sequence which may be predictors of its mechanistic role in the activation/repression of anthocyanin synthesis. The *R* locus MYB protein sequence contains at the N-terminus the R2R3 domains characteristic of the large R2R3-MYB gene family (125 genes) in *Arabidopsis* [10]. Figure 8 shows the conserved amino acids (highlighted in black and gray) in the R2 and R3 domains of the *R* gene MYB protein sequence.

In *Arabidopsis* the 125 MYB proteins of the R2R3 family have been classified into 22 subgroups based on the sequence motifs present towards the carboxy (C)-terminus, downstream of the R2R3 domains that specify their function. One of the subgroups (#5), which includes MYBAN2, MYB75, MYB113 and MYB90, have the KPRPR[S/T]F motif and are regulators of anthocyanin biosynthesis. The *R* locus MYB contains, besides the R2R3 domains, a variant of the subgroup 5 functional motif (**KP**V**PR**AT) (Figure 8A highlighted green and red type). Similarly, other plant species MYB TF with anthocyanin-activating function have variations of that subgroup 5 motif ([R/K]Px[P/A/R]xx[F/Y]) [19]. *Arabidopsis* R2R3-MYB subgroup 4 (AtMYB3, AtMYB4, AtMYB7, AtMYB32) is defined by a C-terminal amino acid motif (LNL[E/D]L) predicted to be involved in transcriptional repression [20], [21], [22], [10]. With the exception of one amino acid this motif is conserved at the C-terminus of the *R* locus R2R3-MYB amino acid sequence (**L**N**L**[E/**D**]**L**) (Figure 8A, fuchsia highlight).

Another molecular feature present in both those MYB subgroups (5 and 6) in *Arabidopsis* is the conserved amino acid signature ([DE]Lx_2_[RK]x_3_Lx_6_Lx_3_R) shown to be the structural basis for the interaction between many higher plants MYB and R/B-like bHLH proteins [23]. This signature is also present in the *R*-locus encoded MYB sequence (Figure 8A, aqua highlight). Except for one, all the conserved amino acids in the signature and with identical spacing are present in the R3 sequence repeat of the *R*-encoded MYB protein (burgundy type). The interaction between MYB and bHLH proteins has been studied extensively for MYB transcription factors regulating the phenylpropanoid biosynthetic pathways and seems to be conserved throughout the plant kingdom [24], [21], [25], [26], [27].

Based on the level of fidelity of all the well characterized MYB sequence domains, signatures and motifs, the MYB transcription factor encoded in the *R* locus could belong to the R2R3-MYB subgroup 5 (activation motif) and 4 (repression motif) that associates with an R/B-like bHLH protein to promote or repress the late portion of the anthocyanin biosynthetic pathway (Figure 7).

The defective *r* allele with the “C”-nt deletion in Exon2 truncates the protein prematurely preserving only the R2 domain and 16 amino acids of the R3 domain (Figure 8B). All other conserved motifs including the activation and repression motifs are lost in the resulting truncated peptide if the transcripts are processed and spliced as those transcribed from the wild type *R* gene. Interestingly, our expression data (Table 3) show that the levels of transcripts from both the standard black *R* and brown *r* alleles are about the same levels, thus the truncation of the protein in the *r* allele does not appear to reduce the levels of the low levels of cytoplasmic mRNAs as is the case for other examples of prematurely truncated proteins, presumably from destabilization of the polysomes and rapid degradation of the mRNAs [28]. Thus, the loss of function of the MYB factor in the brown *r* allele is from the C deletion prematurely truncating the protein and not from changes in transcript levels at the locus.

### The 13 kb TgmR* Element Residing in Intron2 of the RM30-R* Stable Black Revertant Allele Is Removed by Intron Processing to Produce a Fully Functional MYB Transcription Factor from a Reduced Level of Transcripts

**Figure 9:**
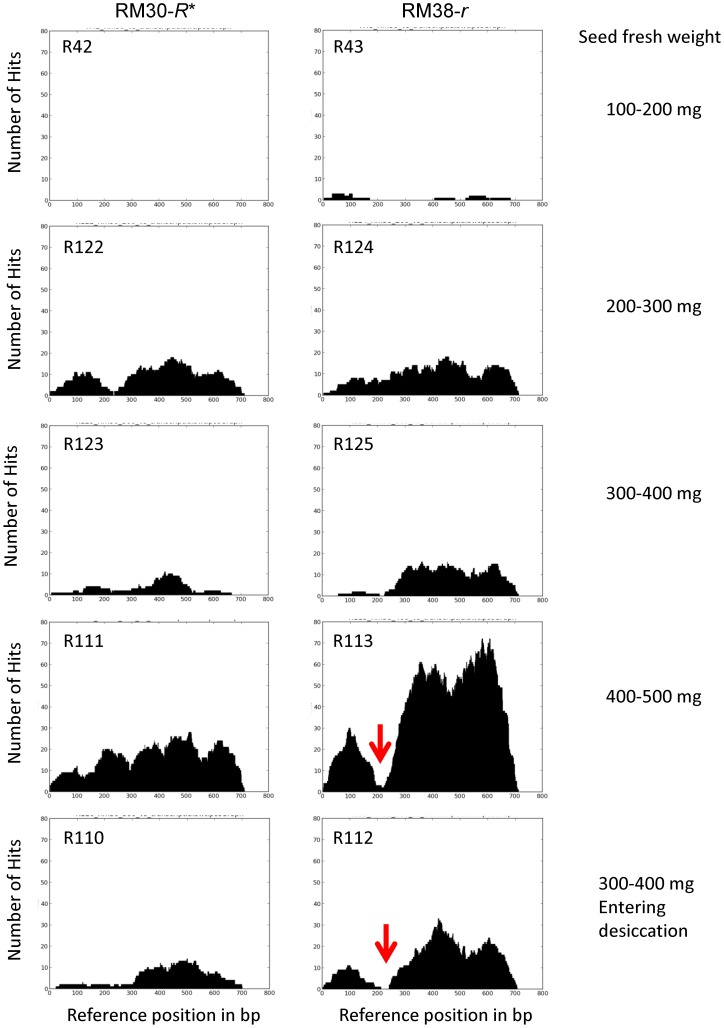
Distribution of RNA-Seq Reads from Seed Coats of the RM30-R* and RM38-*r* When Aligned to the Transcript Sequence of Glyma09g36983. The pair of graphs in each row represent the distribution of non-normalized RNAseq reads that align to the 714 bp transcript sequence from the Glyma09g36983 gene at each of the five stages of seed coat development indicated in the margin to the right (described in Figure 4). The plots in the left column represent the distributions of sequence reads derived from the seed coats of the RM30-R* that is interrupted by the TgmR* transposon and the plots in the right column represent the RNAseq reads derived from the RM38-*r* line with the uninterrupted *R* gene but containing the “C”-nt deletion at 222 bp of the transcript. The numbers of RNAseq reads matching a given position of the *R* gene transcript are plotted against the position along the reference Glyma09g36983 transcript sequence containing the three exons and no introns. No expression was detected in the early 100–200 mg seed developmental stage in the RM30-R* line. The red arrow points to the location of the *r* allele “C”-nt deletion at 222 bp of the transcript.

We inspected the alignment of the RNA-Seq reads from the Rm30-R* and RM38-*r* lines at all five developmental stages (Table 2) against the 714-nt (3-exon gene model) transcript sequence with Bowtie 1. The results presented in Figure 9 show clearly the numbers of sequence reads and their alignments with a total coverage of the Glyma09g36983 gene transcript sequence in both lines. The expression level of the *R* gene is low in black and brown seed coats with the only significant difference manifested in seed coats of the 400–500 mg seeds, with the brown seed coats accumulating slightly more than double the level in the black seed coats (Figure 9) as previously indicated in Table 2 with the RM30-R*/RM38-*r* ratio of 0.49. As stated earlier, these alignment results also show that the Glyma09g36983 gene is transcribed all the way to the 3′end in the RM30-R* black seed coats (Figure 9) despite the large transposon insertion at 110 nt from the beginning of Intron2. These data indicate that despite a very low level of transcripts, they are cleanly processed at the intron exon boundaries.

On the other hand, the alignment of RNA-Seq reads from the RM38-*r* brown seed coats to the 714-nt sequence representing the standard Glyma09g36983 *R* allele transcript, shows a valley observed at 222 bp, the location of the deleted “C”-nt (Figure 9 red arrow). Bowtie 1 does not align any insertion or deletions, thus explaining the lack of alignments at the location of an intron or even a single nucleotide insertion or deletion. These data are additional evidence that RNA sequence reads from the RM38-*r* allele were missing a C at position 222. This conclusion was also directly confirmed by visual inspection showing that sequences aligned properly to either side of the C at nucleotide 222 but none contained the C at position 222. In addition, we performed the alignment of the RM38-*r* RNA-Seq reads to the mutant transcript sequence (with the C deletion) and the valley observed at position 222 in the graph of alignments was no longer present as shown in Figure S1 indicating proper alignment over the full length transcript.

The RNAseq data obtained were also analyzed to determine the level of expression of the putative TgmR* element in order to determine whether there are chimeric read through sequences.

Using the Bowtie 1 alignments, we determined the distribution of sequence reads along the length of the 15,450-nt genomic sequence obtained from the black seed RM30-R* line that comprises the *R* locus gene Glyma09g36983 interrupted by the 13,024-nt TgmR* insertion in Intron2. A representation of the distribution of sequence reads at the five stages of seed development in the black and brown seed lines is shown in Figure S2. It is clear that sequences aligned all through the TgmR* sequence portion in RNA samples from both lines at all five stages of seed coat development examined including a low level from ORF2. Whether these sequences originate from TgmR* or from the other genome regions containing similar elements (Figure 3) cannot be determined from the alignments. Manual inspection of the transcript sequences did not find any chimeric sequences at the intron junctions or the boundaries of the TgmR* element, which again indicates clean processing of the introns from the gene with complete and precise removal of the 13 kb TgmR* element located within Intron2 through the normal intron processing.

### The CACTA Element is Stabilized by Increased Methylation in the RM30-R* Black Seeded Revertant Compared to the Variegated RM55-*r^m^* Mutable Line

The mutable allele at the *R* locus present in the RM55-*r^m^* soybean line which confers the variegated black and brown seed coat phenotype (Figure 1) has the unusual behavior of switching between active and inactive phases somatically and germinally. Heritable changes of this *r^m^* allele were observed in progeny plants that produced plants with all black or all brown seed [6]. Notably, the mutability of the allele resurfaced in progeny plants from the all black (R*/R*) or all brown (r*/r*) revertant seeds. These results indicated that the *r^m^* revertant alleles were not stable and switched between the three forms (*r^m^*, R* and r*) with high frequency.

The genomic sequences of the alleles from the variegated (*r^m^*) and black (R*) seed lines reported here showed that both contained the TgmR* transposon insertion in Intron2. Could the instability of these alleles be dictated by their methylation levels in the two isolines? To answer this question we sequenced the genomes of three soybean lines, RM55-*r^m^* (variegated seed), RM30-R* (black seed) and UC44-*R* (black seed with no TgmR* insertion), using the bisulfite method (BS-seq) first described by Cokus et al, 2008 [29] to yield 226, 209, and 235 million total reads respectively (Table S1). The resulting bisulfite sequences were processed through the three-letter aligner program, Bismark [30] to determine the type and degree of “C”-methylation of the transposon and the upstream (35,513 bp) and the downstream (25,417 bp) regions at the *R* (Glyma09g36983) loci. The results were compiled in Table 4 and revealed a higher degree of methylation for the transposon (TgmR*) sequences, most notably, in the RM30-R* black-seeded revertant line with an almost double (5.8%) level of methylated C in the CHH context compared to 3% in the RM55-*r^m^* (variegated seed) and 2.9% in the UC44-*R* (black seed with no TgmR* insertion). The percentage of CpG methylation in sequences with similarity to TgmR* from both RM30-R* (86.2%) and UC44-*R* (84.7%) were substantially higher than in RM55-*r^m^* (77.6%). CHG methylation in sequences with similarity to TgmR* was also higher for RM30-R* (74.3%) compared to RM55-*r^m^* (63.1%). Although UC44-*R* does not contain TgmR* in the *R* locus, there are highly repeated regions with sequence similarity to the transposon elsewhere in the genome as previously discussed.

**Table 4:** Bismark bisulfite-seq methylation results for the TgmR* CACTA transposon insertion and upstream/downstream regions of Glyma09g36983 in three soybean lines: RM30-R* black, RM55-*r^m^* striped and UC44-*R* black seed coat phenotypes.

DNA reference	Upstream (35,513 bp Glyma09g36983)			TgmR* (13,024 bp in Glyma09g36983)			Downstream (25,417 bp Glyma09g36983)		
Soybean lines sequenced	RM30	RM55	UC44	RM30	RM55	UC44	RM30	RM55	UC44
	(black, R*)	(mutable, *r^m^*)	(black, *R*)	(black, R*)	(mutable, *r^m^*)	(black, *R*)	(black, R*)	(mutable, *r^m^*)	(black, *R*)
No of alignments w/unique best hits	2,678	2,874	3,257	13,134	15,168	15,670	1,895	2,145	2,483
Total methylated C's in CpG context	512	600	678	43,284	45,140	50,347	392	464	650
Total methylated C's in CHG context	144	203	203	50,016	49,405	53,631	85	89	129
Total methylated C's in CHH context	395	256	252	18,596	11,048	10,957	204	134	201
Total unmethylated C's in CpG context	5,649	5,951	6,890	6,943	13,044	9,108	4,265	4,890	5,562
Total unmethylated C's in CHG context	8,510	9,134	10,130	17,341	28,873	27,425	6,129	6,986	7,960
Total unmethylated C's in CHH context	60,635	65,005	73,402	301,746	355,884	369,037	42,634	47,710	55,145
C methylated in CpG context	8.3%	9.2%	9.0%	86.2%	77.6%	84.7%	8.4%	9.6%	10.5%
C methylated in CHG context	1.7%	2.2%	2.0%	74.3%	63.1%	66.2%	1.4%	1.9%	1.6%
C methylated in CHH context	0.6%	0.4%	0.3%	5.8%	3.0%	2.9%	0.5%	0.4%	0.4%

**Figure 10:**
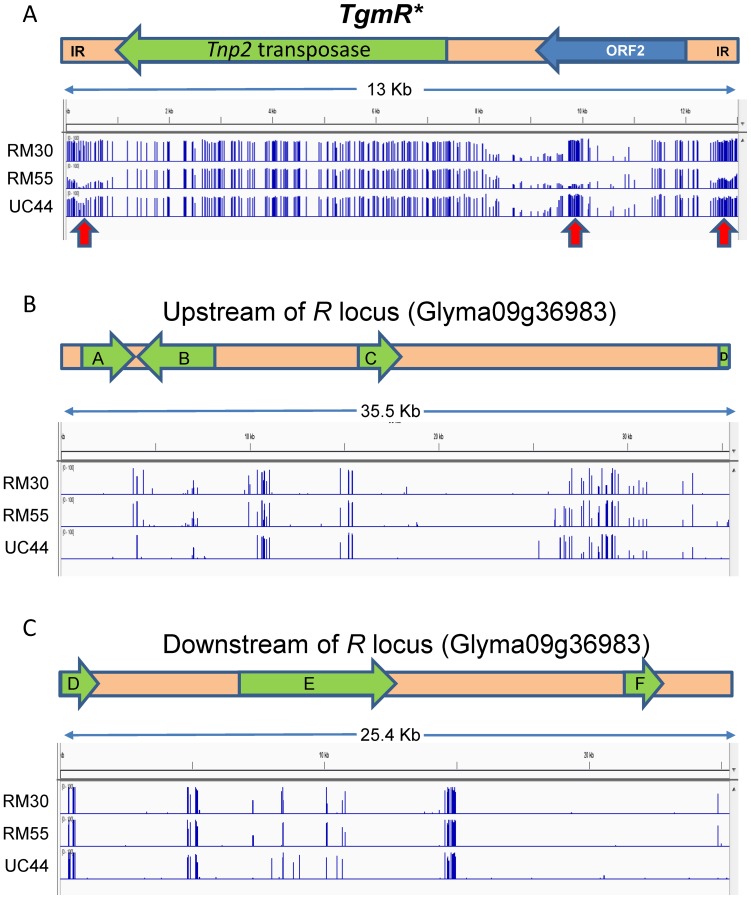
Distribution of Methylation in the TgmR* Transposon (A) and in the Upstream (B) and Downstream Regions (C) of the *R* locus in the Black-seeded RM30-R* and the Mutable RM55-*r^m^* Isolines, and of the UC44 line with a Standard *R* Allele. All graphs were created by the sequence alignment tool of the Integrative Genomics Viewer (IGV) browser. (A) TgmR* methylation regions. The 13 bp CACTA inverted repeat ends and subterminal repeats are marked IR. Lower levels of methylated sequences (marked by red arrows) are found aligning to the subterminal repeats of the TgmR* element and the 3′-end of the predicted ORF2 transcript in the striped mutable RM55-*r^m^* line. The black-seeded RM30-R* line containing the TgmR* insertion and the black-seeded UC44-*R*, line which lacks the TgmR* insertion, present nearly identical patterns of methylation at those three differentiated areas of the TgmR* element. (B) Upstream regions and (C) downstream regions of the TgmR* insertion site in the *R* gene. A few methylation differences appear between the three lines in the upstream and downstream regions, but the most distinct are those for UC44-*R* (Williams self-black seed) which may represent varietal differences. Gene represented by green arrows are: A (Glyma09g36941); B (Glyma09g36950); C (Glyma09g36966); D (Glyma09g36983); E (Glyma09g37000) and F (Glyma09g37010). The upstream and downstream schematics are not drawn to scale.

The distribution of differentially methylated TgmR* regions in the black-seeded RM30-R* and the mutable RM55-*r^m^* soybean isolines is shown in Figure 10A as visualized by the sequence alignment tool of the Integrative genomics viewer (IGV) browser http://www.broadinstitute.org/software/igv/home [31]. Significantly lower levels of methylated sequences appear to align to the terminal repeats of the TgmR* element and the 3′-end of the predicted ORF2 transcript in the striped black/brown-seeded mutable RM55-*r^m^* line. The black-seeded line UC44-*R* line lacking the TgmR* insertion in *R* presents nearly identical pattern and level of methylation in the three areas of the TgmR* as in the RM30-R* line.

The distribution of methylated sequences upstream and downstream of the TgmR* element inserted in the Glyma09g36983 gene for the black-seeded RM30-R* and the mutable RM55-*r^m^* isolines as well as the black-seeded UC44-*R* Williams cultivar are shown in Figures 10B and 10C. A few methylation differences appear between the three lines but the most distinct are those for UC44-*R* which may represent varietal differences and not affect significantly the expression of the genes encoded upstream (A, B, C and D-5′end) and downstream (D-3′end, E and F) of the *R* locus Glyma09g36983 gene. Therefore, the methylation differences for the *r^m^* and R* alleles in the RM55-*r^m^* and RM30-R* isolines seemed to be confined primarily to the TgmR* sequences at its extended terminal repeats and to the 3′end of the ORF2 region.

## Discussion

### Structural Variations in the RM55-*r^m^* and Revertant RM30-R* Alleles Confirm the *R* locus as Glyma09g36983 that Encodes a MYB Transciption Factor

We have previously identified and characterized the expression of several key loci controlling the synthesis of anthocyanins in the soybean seed coats. The *I* locus was found to be a cluster of *CHS* genes that produce small RNAs that downregulate CHS transcripts [2], the *T* locus encodes a flavonoid 3′hydroxylase (*F3′H*) gene [3], and the *Wp* is a flavanone 3-hydroxylase (*F3H*) gene [32] (Figure 7). However, the *R* locus that with *T* determines the various colors of the seed coat in genotypes with a recessive *I* allele (*i*) is not completely understood. Recently, it was proposed that *R* maps to a locus, Glyma09g36983, encoding a MYB transcription factor that may positively regulate the expression of a *UF3GT* gene, which catalyzes the glycosylation of cyanidins to anthocyanins in the last step of anthocyanin synthesis [4]. However, another report suggested a different transcription factor location 120 kb away on the same chromosome [5].

The results we present here support the location of the *R* locus to be the MYB encoded by Glyma09g36983 as determined recently by Gillman et al., 2011 [4] based on mapping polymorphisms associated with standard *R* and *r* alleles, one of which was a “C” deletion that truncates the MYB protein in the standard brown *r* allele. Using primers designed to Glyma09g36983, our analysis of two isolines including a mutable RM55-*r^m^* line with black and brown striped seed phenotype and RM30-R*, a stable black-seeded revertant isoline, revealed the existence of a 13 kb CACTA transposon insertion (TgmR*) at the beginning of the Glyma09g36983 gene Intron2 in both of these lines (Figures 2 and 3). The transposon was absent in the brown-seeded RM38-*r* line. Thus, our data directly implicate the structural variations in Glyma09g36983 as producing the phenotypic differences of the *R* locus.

### The TgmR* Sequence Confirms the Existence of Two CACTA Subfamilies in Soybean

Based on differences in the subterminal repeats, we previously predicted the existence of two CACTA subfamilies in soybean [8]. The novel TgmR* element is 13 kb in length and has all the characteristic features of a CACTA transposon based on the three base duplication (ATG) at the site of insertion, the CACTA ends and the direct and reverse repeats at the subterminal ends that can fold into complex secondary structures (Figure 3A, Files S2 and S3). TgmR* confirms the proposed existence of two subfamilies of CACTA transposons in soybean which was based on the two distinct types of repeated motif at the subterminal ends (Figure 3B). The motif in TgmR* is more closely related to the one in *Tgm1*, a defective element interrupting a soybean seed lectin gene [7], [14], than that of three other previously characterized elements (Tgmt*, Tgmw4m, Tgm-Express1) [32], [8]. In addition, the TgmR* 13 kb element encodes two genes, ORF1 (Tnp2-like transposase) and ORF2 of unknown function. The transposase region of TgmR* is only 71% similar to the transposase in Tgmt* (*Tgm9*). Thus, we propose that TgmR* is an active element of the *Tgm1* type family of elements. Based on RNA-Seq data, both ORFs of TgmR* or related element are expressed at low levels in the seed coats of both black RM30-R* and RM38-*r* lines (Figure S2). Because DNA sequences with high similarity to the ORF1 gene are highly repeated throughout the entire genome, the ORF1 RNAs detected in both lines could be derived from sequences other than the one inserted in the R* allele.

### The Revertant RM30-R* Allele Has Black Seed Coats as its Intron2 with the TgmR* Insertion is Correctly Spliced to Produce Functional MYB Transcripts

The RNA-Seq analysis at multiple stages of seed development was crafted to determine whether the TgmR* insertion interfered with the expression of the *R* (Glyma09g36983) gene. We contrasted the normalized RPKM reads from the black RM30-R* revertant line to those of the brown RM38-*r* line that aligned to the Glyma09g36983. The results were congruent with TgmR* having a negative effect on the expression of the R* allele, since a higher number of RNAseq reads were found to align to Glyma09g36983 from the RM38-*r* line than from the RM30-R* line at late stages of seed development (Table 2, Figures 4 and 9). The level of expression was very low in both lines, but this is common for other transcription factors. The difference in *R* expression between the two lines is more marked at the 400–500 mg seed developmental stage with a RM30-R*/RM38-*r* ratio of 0.49 and a p-value of 0.069 (Table 2).

These results were not surprising since the 13 kb TgmR* insertion in the R* allele of the RM30-R* line could reduce the level of its transcription. However, based on alignments of the RNA-Seq reads from RM30-R* to the *R* Glyma09g36983 transcript sequence shown in Figure 9, the TgmR* element appears to be cleanly removed with the correct intron processing as there was full coverage of the entire transcript with the aligned reads.

In contrast, a deep valley at around 200 bp occurs in the RM38-*r* seed coats (Figure 9), which suggests the absence of RNA-Seq reads that align to that region. The region of non-alignment occurs because of the “C”-nt deletion at 222 bp in the RM38-*r* allele. The transcripts from the *r* allele of the brown-seeded line (RM38-*r*) with the “C”-nt deletion in Exon2 also reach full length. If the processing of the *r* allele transcripts occurs at the same exon-intron splice junctions, the translation product of this defective *r* allele will be terminated prematurely eliminating the activation (KPRPR[S/T]F), repression (LNL[E/D]L), and bHLH (DLx_2_ Rx_3_Lx_6_Lx_3_R) binding domains (Figure 8), rendering the transcription factor non-functional resulting in brown seed coats.

### The MYB Factor Upregulates *ANS* Genes in the Anthocyanin Biosynthesis Pathway Late in Seed Coat Development

Although many of the genes in the anthocyanin pathway examined showed differences in expression between the RM30-R* black revertant and the RM38-*r* brown isoline, the most significant differences detected were for the *ANS2* and *ANS3* genes at the 400–500 mg seed developmental stage (Table 2, Figure 6A). In another study in which the transcript profiles of seed coats from a standard black and brown-seeded near-isogenic pair were compared at different stages of seed development, significant differences in *ANS2/ANS3* and *UGT78K1* expression were also seen between the two isolines with higher levels in the black seed coats [5]. For *ANS2/ANS3,* the significant differential expression (P<0.01) manifested early in seed development, (75–100 mg), while the *UGT78K1* differences were first detected at 200–300 mg weight range and reaching maximum at 300–400 mg seed size. Gillman et al, (2011) [4] measured *ANS* and *UF3GT* expression via qRT-PCR in seed coats of a brown- (PI 567115 B) and black- (PI 84970) seeded lines with similar results. As in those instances, our results show that the *UFGT* genes are expressed at very low level at early stages of seed development, while *ANS* expression is very high at those early stages and tapers down as seed development advances. This decrease is slowed down or reversed in the black-seeded phenotypes. The higher level of *ANS* gene expression at early stages of seed development can be explained by an additional role of the ANS enzyme in directing the synthesis towards the flavanol and proanthocyanidin branches of the flavonoid pathway (Figure 7). In contrast, the UFGT enzyme function is limited to the last step in the synthesis of anthocyanins (Figure 7).

Whether the MYB transcription factor encoded at the *R* locus regulates all or one of the genes in the anthocyanin pathway is not clear from the available data. A search in MEME Version 4.9.1 [33] for MYB transcription factors binding motifs in the 2-kb upstream sequence of each of seven genes from the anthocyanin pathway found two motifs in all seven genes. One of those motifs is a MYB binding site (JASPAR_CORE_2014) for Arabidopsis R2R3 MYBs (AtMYB84, AtMYB15, AtMYB77) of subgroups other than number five that contain the activation motif KPRPR[S/T]F.

The structural genes of the flavonoid metabolic pathways are regulated for the most part, at the transcriptional level and, with some exceptions, the majority of the regulatory genes have been found to be members of a large family of MYB transcription factors. Most of the MYB genes were predicted to function as transcriptional activators of the flavonoid biosynthetic pathway but in a few instances transcriptional repression guided by MYB transcription factor has been demonstrated [21]. In maize, *C1-I* was found to be a dominant inhibitor allele of the transcription activator *C1-myb-*like gene [34], [35]. A different *C1* homologue of maize, *Zm38*, was found to be structurally similar to two other R2R3 MYB genes, *AmMYB308* and *AtMYB4*, that are transcriptional repressors of *4Cl* (b-coumaroyl 4-CoAligase) and the *C4H* (cinnamate 4-hydroxylase) genes in *Antirrhinum* and *Arabidopsis*, respectively [36], [22]. Another TF with the motif shown to be involved in repression of transcription in *Arabidopsis* (*AtMYB4*) is the *FaMYB1* gene expressed during fruit maturation in strawberry [21]. *AmMYB308*, like *C1-I*, competes out and inhibits the action of their respective transcriptional activator alleles. In contrast, *AtMYB4* and *FaMYB1* are direct repressors of their respective target genes and contain sequence motifs that recognize specific promoter repressor domains. Although little is known about repression mechanisms in plants, in both those instances, AtMYB4 and FaMYB1 C-termini possess a peptide motif, pdLNL^D^/_E_Lxi^G^/_S_, shown to be required for suppression activity [22], [21], [20]. The soybean *R* locus MYB factor has the core portion of such motif, **LLDL**, at its C-terminus which may suffice to function as a direct repressor of late genes in the anthocyanin biosynthetic pathway. Likewise, the *R* gene possesses four of the amino acids in the motif, **KP**R**PR**[S/T]F, present in the subgroup five (AtMYB113) involved in anthocyanin regulation by activation [10]. The differential expression of the *R* MYB gene and *ANS* genes supports its role as an inducer of anthocyanidin synthase in the black soybean seed coats.

Our results show that the Intron2 with the TgmR* insertion is spliced out of the R* allele transcripts correctly allowing a small amount of the MYB to be translated in sufficient levels to induce the transcription of *ANS2/ANS3* and *UFGT* whose enzyme products will complete the last two steps of anthocyanin synthesis.

The RNAseq expression results also demonstrate that the activation of the anthocyanin pathway in RM30-R* is due to the Glyma09g36983 with the TgmR* transposon and not to either of the two other closely related MYB transcription factor genes, Glyma09g36966 and Glyma09g37010, since these two genes appear not to be expressed in the seed coats of either the RM30-R* line with the black seed and the RM38-*r* line with the brown seed phenotypes. This eliminates the likelihood that the TgmR* insertion occurred in any of those two related genes that could in turn affect the expression of the Glyma09g36983 gene in a similar fashion as the *C1* homologue of maize, *Zm38*, that was found to be a transcriptional repressor of *C1* [37].

### Hypomethylation is Associated with the Variegated RM55-*r^m^* Mutable Allele Compared to the Stable RM30-R* Black Revertant

The soybean line with the mutable *r^m^* allele (RM55-*r^m^*) and striped seed phenotype showed instability with the frequent generation of revertant seed genotypes some being all black which in subsequent generations would switch back to variegated at a low frequency [6]. To find out whether methylation contributes to the variegated or stable phenotype, we compared the BS-sequences from the striped RM55-*r^m^* and the stable revertant isoline RM30-R*. As shown in Table 4, the level of methylated C residues in the CHH context for TgmR* in the RM30-R* stable line was double the percentage of that found in RM55-*r^m^*. The more prevalent methylated CpG and CHG regions were also higher in comparison to RM55-*r^m^*. More interestingly, distribution of the hypomethylated regions in the element were concentrated near the subterminal repeats and a region of the 3′ end of ORF2 (Figure 10).

### The RM30-R* and RM55-*r^m^* Alleles resemble Maize *Spm*-Suppressible Alleles that are Responsive to the Methylation States of the Element

Involvement of methylation causing the variegated phenotype is consistent with the observation that there appear to be no structural changes in the MYB gene when the element excises from Intron2 since the 929-bp amplicon from the RM55-*r^m^* DNA aligned with the Glyma09g36983 gene sequence in Phytozome (Williams 82 which has a standard *R* allele) with no rearrangement of Exon2 or the Exon2-Intron2 splice site junction. This suggests that when the element excises by transposition, a fully functional MYB protein is produced leading to the black sectors on the brown background in the RM55-*r^m^* line.

The brown background in the RM55-*r^m^* seed coats being the result of the 14 kb amplicon 13 kb TgmR* (ie the 14kb amplicon) is harder to explain especially in light of the fact that we have shown that the revertant RM30-R* line containing the 13 kb TgmR* element in its intron has black seed coats. This would suggest that the phenotype of the allele (black or brown) is determined by the level of methylation of the TgmR* element. When the element residing in Intron2 is less methylated as in the RM55-*r^m^*, the sectors are brown implying the MYB gene is not expressed. When TgmR* is more methylated, as in the RM30-R* allele, the seed coats are black and the MYB gene is expressed as we have shown experimentally. We propose that the mechanism for this phenenomon is similar to that shown for the *Spm*-suppressible alleles of maize in that the phenotype of the affected allele can depend upon where the transposon sits in the gene and whether an active element is present (reviewed in [12], [13]). The CACTA ends of *Spm* elements as well as the Activator (Ac) elements sometimes mimic splicing sites and are recognized as intron ends [12], [38]. In some cases as shown in maize, binding of transposase from an active element will interfere with intron processing if the element is near the Exon-Intron junction leading to the gene not being processed correctly. In the absence of transposase, the introns are processed and the gene is expressed, although sometimes at a lower level. In maize, it has been shown that the autonomous *Spm* element itself can switch between an active state (less methylated with transposase produced) and an inactive state (more methylated and transposase not produced) based on the level of its methylation [11]. Thus, in the hypomethylated RM55-*r^m^* line, transposase from the TgmR* element in the MYB gene or other locations could bind to the element ends and interfere with intron processing leading to the brown background color. On the other hand, in the stable black line RM30-R*, the opposite effect occurs as the element is more methylated and transposase is lacking or cannot bind the element termini and interfere with intron processing. Thus, the element is spliced out with the intron and the MYB gene is expressed at a low level leading to activation of the ANS genes and production of the anthocyanin pigments. There is one phenotypic difference between the standard *R* allele and the R* allele in the RM30 line in that the hilum region, which is the point of attachment of the seed to the pod, is less pigmented than in the standard *R* allele and retain a brownish cast [6]. A genetic difference between the two alleles is that the *r^m^* is normally recessive to the standard *R* allele producing progeny with black seed, but it appears to be dominant to the R* allele with variegated seed predominating and an increased rate of somatic and germinal mutability results from crossing RM30-R* and RM55-*r^m^* [39]. The activation *in trans* of the TgmR* element within the RM30-R* allele by the transposase from the hypomethylated *r^m^* allele is consistent with its dominance over the R* allele.

## Summary

In conclusion, our results confirm the soybean *R* locus to be Glyma09g36983 and demonstrate that the transcription of the *R* gene in the black seed coats of the RM30-R* revertant line, may be hindered by the processing of the very large Intron2 containing the TgmR* (13kb) insertion, but it does not render the processed transcript non-functional. Instead, the translated MYB protein drives an increase in transcript accumulation of the *ANS2/3*, *UFGT2* and *AOMT* genes whose products function in the late steps of anthocyanin synthesis, particularly in production of the cyanidin-3-glycoside that accounts for the black pigmentation of the seed coats. In contrast, the observed higher level of transcripts found for the defective *r* allele in RM38-*r* seed coats may translate into truncated peptides that are non-functional and do not activate the transcription of the late anthocyanin pathway genes resulting in brown seeds which reflects accumulation of only proanthocyanidins. The TgmR* found in the striped RM55-*r^m^* and black-seeded RM30-R* isolines is a transposon of a second CACTA subfamily that encodes a full length *Tnp2* transposase gene that may drive the observed instability of the isolines with the *r^m^* and R* alleles. The level of instability of the RM55-*r^m^* and RM30-R* isolines is influenced by the total level of C methylation and also methylated C in the CHH context, judging by the bisulfite sequencing results of three genomes. The TgmR* element in the variegated RM55-*r^m^* line was hypomethylated especially at the subterminal repeat ends compared to the RM30-R* element. Stress and other environmental factors may in turn affect the methylation level of the germinal and somatic tissues and consequently lead to the variegated phenotype and affect the stability of the mutable (*r^m^*) and revertant (R*) alleles by a mechanism similar to that elucidated for CACTA elements in maize including the *Spm*-suppressible alleles first described by Barbara McClintock (reviewed in [12], [13]). This explains the opposite phenotypes resulting from the influence of the TgmR* transposase on intron processing of the MYB protein when the element is less methylated in the RM55-*r^m^* line (brown coat sectors) versus the more methylated element in the RM30-R* revertant line with black seed coats.

## Materials and Methods

### Plant Material and Genotypes

The *Glycine max* cultivars and isolines used for this study, including their genotypes and phenotypes, are described in Table 1. All lines are homozygous for the indicated alleles at the *R* locus, and only one of the alleles at each locus is shown for brevity in the table and text. Plants were grown in the greenhouse and shoot tips (meristems surrounded by the primordial leaves) and seed coats dissected from seeds at varying stages of seed development were frozen in liquid nitrogen, freeze dried (VirTis *Benchtop* K) and stored at −20°C. The seed coats used for this study were those of seeds with fresh weight of the whole seed ranging from 100–500 mg. An additional, more advanced developmental stage used was from seeds with 300–400 mg fresh weight that had entered the dehydration process (Figure 4).

### DNA Extractions and Long Range (LA) PCR Reactions

Genomic DNA was isolated from freeze dried shoot tips dissected from two weeks old soybean plants using a microprep method [40] with minor modifications [3].

Based on the Glyma09g36983 genomic sequence from Phytozome, multiple DNA oligo-primer pairs were designed to be use in PCR reactions that could amplify the Glyma09g36983 gene in all three soybean lines (RM30, RM38 and RM55) with the mutable and stable *R* loci. The DNA oligo-primers were synthesized at IDT (Integrated DNA Technologies) and the name and sequence of one forward and two reverse oligo-primers that successfully amplified a large portion of the *R* alleles in all the lines are:

FP: R6990FP1 (GAGTTGAAGGAATTGAGTTATATACGTACACCTGAAC) (37 bases)

RP: R6990RP1 (CATCGTATAAAACCTTTATGCTGTCCATGTC) (31 bases)

RP: R6990RPB (CAACGACAACAGTCATAATGACGGTGATGATAACAG) (36 bases).

The PCR reaction conditions used to successfully amplify the transposon insertion at the left border of Intron2 were the following: Initial denaturation step at 94°C for 2 min followed by 30 cycles of 94°C for 30 sec denaturation, 1 min annealing at 68°C and 9 min extension at 68°C. This was followed by a 10 min extension at 72°C. The reagents for the reactions were those provided with the *TaKaRa LA Taq* Hot Start Version kit No. RR042A (TaKaRa Bio Inc, Dalian, CO). DNA template was 1 µg in 50 µl reaction. The amplification fragments were separated in a 0.8% preparative SeaPlaque Agarose (Lonza Rockland, ME) gel in 1x TA buffer. The large ∼14 kb and smaller 929-nt amplified DNA fragments were extracted and cleaned from the gel pieces using The Zymoclean Large Fragment DNA Recovery Kit (#D4045) (Zymo Research Co) according to their protocol. The cleaned larger ∼14 kb DNA fragments were sequenced via the 454 method while the smaller 929-nt fragments were sequenced using the Sanger method at the W. M. Keck Center for Comparative and Functional Genomics at the University of Illinois at Urbana-Champaign.

### Sequencing on the Roche GS-FLX+ System

Two ∼14 kb PCR fragments amplified with two oligomer primers (R6990FP1 and R6990RPB, sequences shown above) from the mutable RM55-*r^m^* and revertant RM30-R* soybean lines were submitted to the W. M. Keck Center for Comparative and Functional Genomics at the University of Illinois at Urbana-Champaign. A shotgun genomic DNA library was prepared from each PCR sample using the Roche GS Rapid Library Prep Kit following the Roche Rapid Library Preparation Manual instructions (Roche Applied Sciences, Indianapolis, IN), with the exception that the sheared PCR products were size selected on agarose gel from 600–1000nt after sonication. The final libraries were quantitated using a Qubit fluorometer (Invitrogen, CA) and average fragment sizes were determined by analyzing 1 µl of the library on the Bioanalyzer (Agilent, CA) using a High-Sensitivity DNA LabChip (shotgun library).

Libraries were pooled evenly and diluted to 1×10^6^ molecules/µl for sequencing. Emulsion-based clonal amplification and sequencing on the 454 Genome Sequencer FLX+ system (400 flow cycles) were performed according to the manufacturer's instructions (454 Life Sciences, Branford, CT). The pool was sequenced on two 1/16^th^ lanes of a 70×75 PicoTiter Plate with the Roche XL+ sequencing kit (454 Life Sciences) with software version 2.8, flow pattern B. Amplicon signal processing and base calling were performed using the bundled 454 Data Analysis Software version 2.8.

The DNAs from the two PCR fragments were barcoded (RM30: #11; RM55: #12) and pooled in the two 1/16 lanes for sequencing. A total of 36,359 sequence reads with an average length of 507 nt were obtained from the two 1/16 lanes after quality trimming. The sequence reads were assembled into contigs using the 454 Sequencing System Software V.2.6.

### Construction of Shotgun Genomic DNA Libraries and Sequencing on the Illumina HiSeq2000

Shotgun genomic DNA libraries were constructed using the TruSeq DNA Sample prep kit (San Diego, CA). Briefly, 1 μg of genomic DNA from shoot tips was nebulized at 32 psi for 1 minute. After nebulization, DNA was blunt-ended, 3′-end A-tailed and ligated to indexed adaptors. The adaptor-ligated genomic DNA was amplified by PCR to selectively enrich for those fragments that have adapters on both ends. The libraries were loaded onto 25 Ex-Gels (Life Technologies, CA) and the fraction 600–800 bp was excised from the gel. The final libraries were quantitated by qPCR on an ABI 7900. Final amplified libraries are also run on Agilent bioanalyzer DNA 7500 LabChips (Agilent, Santa Clara, CA) to determine the average fragment size and to confirm the presence of DNA with the expected size range.

The libraries were multiplexed and loaded onto 8-lane flowcells for cluster formation and sequenced on an Illumina HiSeq2000. The DNAs in these libraries were sequenced from both ends of the molecules to a total read length of 100 nt from each end using a TruSeq SBS sequencing kit version 3. The run generated raw basecall files (.bcl) which were converted into demultiplexed compressed fastq files using Casava 1.8 (Illumina, CA). The total numbers of read counts of approximately 200 million at each end are shown in Table S1.

### RNA Extraction, High-throughput RNA Sequencing and Alignment to *G. max* Gene Models

Total RNA was isolated from seed coats of seeds at five stages of seed development using a phenol-chlorophorm and lithium chloride precipitation method [41] that was modified to prevent RNA adhesion to procyanidins (polyphenols) [17], [18].

The purified RNA samples were sequenced at the W. M. Keck Center for Comparative and Functional Genomics at the University of Illinois at Urbana-Champaign. Starting with 1 µg total RNA per sample, RNAseq libraries were constructed with the TruSeq RNA Sample Preparation Kit (Illumina San Diego, CA). These libraries were multiplexed and loaded onto 8-lane flowcells for cluster formation and sequenced on an Illumina HiSeq2000. One of the lanes was loaded with a PhiX Control library that provides a balanced genome for calculation of matrix, phasing and prephasing, which are essential for accurate basecalling. The libraries were sequenced from one end (single-reads) of the molecules to a total read length of 100-nt.

The sequencing run generated.bcl files that were converted into demultiplexed compressed fastq files using Casava 1.8.2 (Illumina, San Diego, CA). A secondary pipeline decompressed the fastq files, generated plots with quality scores using FastX Tool Kit, removed perfect matches to reads that contain only adaptor and generated a report with the number of reads per sample/library.

The resulting sequence reads were aligned to the 78,773 Glyma1 cDNA soybean gene models determined by the Soybean Genome Project, Department of Energy, Joint Genome Institute (Schmutz et al, 2010), using the alignment program Bowtie [42]. Bowtie parameters allowed up to three mismatches and up to 25 alignments per read; reads aligned more than 25 times were discarded, with none of their alignments added to the final count. The total read counts, generally from 30 to 50 million per sample at shown in Table S1.

### Bisulfite (BS) Sequencing

The shotgun DNA libraries were prepared at the W. M. Keck Center for Comparative and Functional Genomics at the University of Illinois at Urbana-Champaign with the Library Construction kit from Kapa Biosystems with one modification: after adaptor ligation, libraries were treated with the EZ DNA Methylation-Lightning kit (Zymo Research). Bisulfite-treated libraries were amplified with the Kapa HiFi Uracil+ DNA Polymerase. The libraries were quantitated by qPCR and each library was sequenced on one lane for 101 cycles from each end of the fragments on a HiSeq2000 using a TruSeq SBS sequencing kit version 3. The average DNA fragment size was 500 bp (ranging from 350 bp to 700 bp) and the sequence reads were 100-nt in length. Fastq files were generated with the software Casava 1.8.2 (Illumina). The total number of read counts of approximately 209, 226, and 234 million for the three samples are shown in Table S1.

The sequence of adaptors used to make the libraries were: (AGATCGGAAGAGCACACGTCTGAACTCCAGTCACNNNNNNATCTCGTATGCCGTCTTCTGCTTG (NNNNNN = 6 nt index)) for adaptor sequence in read 1, and (AGATCGGAAGAGCGTCGTGTAGGGAAAGAGTGTAGATCTCGGTGGTCGCCGTATCAT) for adaptor sequence in read 2.

The Integrative genomics viewer (IGV) browser was used to visualize the methylated C-nt in alignments to the genomic sequences of the R* locus and surrounding upstream and downstream sequences (http://www.broadinstitute.org/software/igv/home) [31].

### Sequencing Data Values, Clustering of Sequences and Gene Model Annotations

The RNAseq results are given in reads per kilobase of gene model per million mapped reads (RPKMs) [43]. The total number of mapped reads, allowing up to 3 mismatches, takes into account reads that aligned up to 25 different times, including multiple segment matches within the same gene model using Bowtie v1 [42]. Annotations of gene model were obtained from the Soybean Genome Project in the Phytozome database version 9.0 [44] with Gmax_109 genome or Gmax_189 and other previously characterized *G. max* cDNAs that have been entered in GenBank.

### Phytozome Glyma Models Corresponding to Various Anthocyanin Pathway Genes, GenBank Accession Numbers and Information Sources

The choices of Glyma models representing the different genes of the anthocyanin pathways and listed in Table 2 were based on previous gene characterization studies. Here we provide the Glyma models, GenBank accession numbers and references for each one of the genes whose expression we analyzed in the soybean lines used for the current study. The *I* locus (Glyma01g43880.1) *chalcone synthase 7* (*CHS7*) Ac.N.: M98871 [45] and (Glyma11g01350.1) *CHS8* Ac.No.: AY237728; Glyma20g38560.1 *chalcone isomerase 1A* (*CHI1A*) Ac.No.: AY595413 [46] and Glyma20g38580.1 (*CHI2*) Ac.No.: AY595415 [46]; *T* locus (Glyma06g21920.1) *flavonoid 3′hydroxylase* (*F3′H*) Ac.No.: EU190438 [3]; *W1* locus (Glyma13g04210.1) *flavonoid 3′-5′-hydroxylase* (*F3′5′H*) Ac.No.: EF174665 [47]; *Wp* locus (Glyma02g05450.1) *flavanone 3-hydroxylase* (*F3H*) Ac.No.: AY669325, [47]; *W3* locus (Glyma14g07940.1) *dihydroflavonol-4-reductase 1* (*DFR1*) Ac.No.: AF167556; *W4* locus (Glyma17g37060.1) *DFR2* Ac.No.: EF187612 [9]; Glyma01g42350.1 *anthocyanidin synthase 2* (*ANS2*) Ac.No.: AY382829.1 and Glyma11g03010.1 (*ANS3*) Ac.No.: AY382830; Glyma08g07130, *UDP-glucose:flavonoid 3-O-glucosyltransferase* (*UF3GT*) (*UGT78K2*) Ac.No.: HM591298.1 [5] and Glyma07g30180 (*UGT78K1*) Ac.No.: GU434274.1 [48]. The latter, *UGT78K1*, was found to be expressed in the cotyledons in addition to the seed coats [4]. Glyma08g06630.1, *anthocyanidin reductase* (*ANR1*) Ac.No.: NM_001254984.1 and Glyma08g06640.1 (*ANR2*) (LOC100817081) Ac.No.: NM_001256143.1 [49]. The *ANR* genes function in diverting the anthocyanidins towards the synthesis of proanthocyanidins (tannins) which provide the color to the brown seed coats (Figure 1). Lastly, Glyma05g36210.1, *anthocyanin O-methyltransferase* (*AOMT*) Ac.No.: NM_001255526.1 [5] which methylates the anthocyanins.

## Supporting Information

Figure S1 Distribution of RNA sequence reads expressed in seed coats of 400 mg seed in the RM38 line with the defective *r*-allele when aligned to (A) the wild type *R*-gene transcript sequence and (B) the mutant *r*-allele transcript lacking the “C”-nt at position 222.
(PPTX)

Figure S2 Distribution of RNA-Seq Reads from Seed Coats of the RM30-R* and RM38-*r* when Aligned to the Genomic Sequence of Glyma09g36983 with the TgmR* Insertion in Intron2.
(PPTX)

File S1 Glyma09g36983 DNA Sequence and Map Locations of Oligonucleotide Primers.
(DOCX)

File S2 TgmR* DNA Sequence with Highlighted ORFs and Terminal End Repeats.
(DOCX)

File S3 TgmR* Terminal Direct Repeats.
(DOCX)

Table S1 Summary of Sequence Reads from 454, Genomic DNA, Methylation and RNA-Seq Sequenced Libraries Made from RM30-R*, RM55-*r^m^*, and RM38-*r* Soybean Lines.
(DOCX)
