# Supplementary material for: Methylation Affects Transposition and Splicing of a Large CACTA Transposon from a MYB Transcription Factor Regulating Anthocyanin Synthase Genes in Soybean Seed Coats
Source: PLoS One. 2014 Nov 4;9(11):e111959. doi: 10.1371/journal.pone.0111959 (PMC4219821; doi:10.1371/journal.pone.0111959)
Supplement: Figure S1 — Distribution of RNA sequence reads expressed in seed coats of 400 mg seed in the RM38 line with the defective r-allele when aligned to (A) the wild type R-gene transcript sequence and (B) the mutant r-allele transcript lacking the “C”-nt at position 222. (PPTX) [file pone.0111959.s001.pptx]

## Slide 1
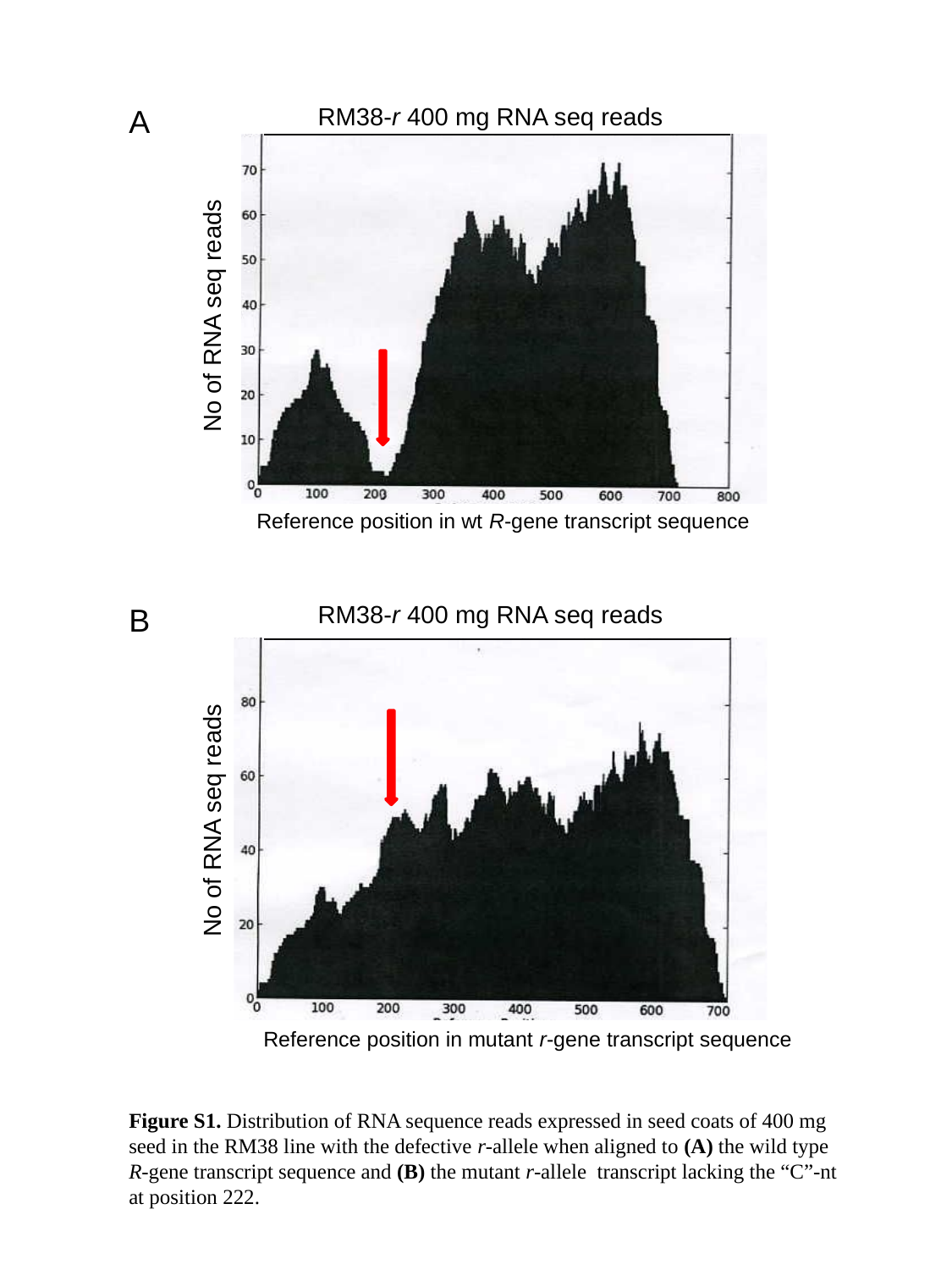

RM38-r 400 mg RNA seq reads
A
No of RNA seq reads
Reference position in wt R-gene transcript sequence
RM38-r 400 mg RNA seq reads
B
No of RNA seq reads
Reference position in mutant r-gene transcript sequence
Figure S1. Distribution of RNA sequence reads expressed in seed coats of 400 mg seed in the RM38 line with the defective r-allele when aligned to (A) the wild type R-gene transcript sequence and (B) the mutant r-allele transcript lacking the “C”-nt at position 222.
