# Supplementary material for: Methylation Affects Transposition and Splicing of a Large CACTA Transposon from a MYB Transcription Factor Regulating Anthocyanin Synthase Genes in Soybean Seed Coats
Source: PLoS One. 2014 Nov 4;9(11):e111959. doi: 10.1371/journal.pone.0111959 (PMC4219821; doi:10.1371/journal.pone.0111959)
Supplement: Figure S2 — Distribution of RNA-Seq Reads from Seed Coats of the RM30-R* and RM38-r when Aligned to the Genomic Sequence of Glyma09g36983 with the TgmR* Insertion in Intron2. (PPTX) [file pone.0111959.s002.pptx]

## Slide 1
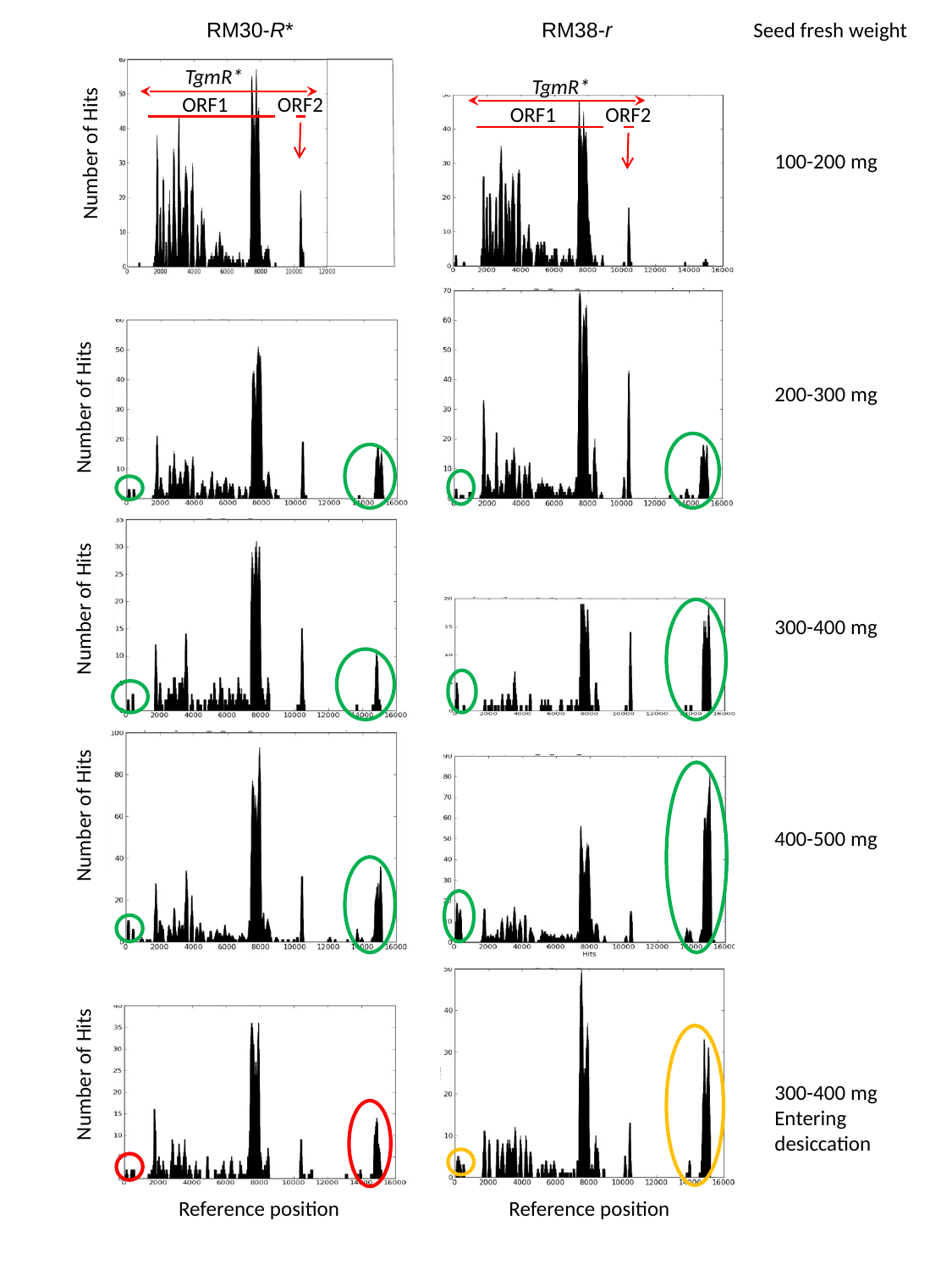

RM30-R*
RM38-r
Seed fresh weight
TgmR*
TgmR*
ORF1
ORF2
ORF1
ORF2
Number of Hits
100-200 mg
200-300 mg
Number of Hits
Number of Hits
300-400 mg
Number of Hits
400-500 mg
Number of Hits
300-400 mg
Entering
desiccation
Reference position
Reference position
