# Supplementary material for: Methylation Affects Transposition and Splicing of a Large CACTA Transposon from a MYB Transcription Factor Regulating Anthocyanin Synthase Genes in Soybean Seed Coats
Source: PLoS One. 2014 Nov 4;9(11):e111959. doi: 10.1371/journal.pone.0111959 (PMC4219821; doi:10.1371/journal.pone.0111959)
Supplement: File S1 — Glyma09g36983 DNA Sequence and Map Locations of Oligonucleotide Primers. (DOCX) [file pone.0111959.s003.docx]

**File S1**

**Glyma09g36983 DNA Sequence (Gm09:42,562,649..42,564,660).** Confirmed by cDNA sequence (Gillman et al. 2011).

**[Glyma09g36990 (Gm09:42,562,649..42,564,493), Phytozome v1.0 transcript]**

GAAAGAACTCTCAGGAGAGGGTGTGGTAATTTTGGCAGTAAGCTGGTATATATACGTTTTCTCCATAAATTGTTAGATACGTGTGTCTCATTTAATACGG

**R6990FP1 (37 bp) 🡪**
TAGCTAATTATACTTACTTTAAA GAGTTGAAGGAATTGAGTTATATACGTACACCTGAACAAAATTAATGTAGCAAATTAACTTATAAGCACCTGGTCTC
ATGGAAGGATCATCAGGTGTGAGGAAAGGCACATGGAGTCAAATTGAAGATGATCTTCTCAAAGCTTGCGTGCAACTTTATGGGGAAGGAAATTGGCACC
TTGTTCCTAAAAGAGCAGGTGTACTTCTAGTTAAATTTTATTTTTTTTTTCGTTTTCTTCATTGCTTGTGATTGCAATGATATGATATAATTACATAACT
ATGAGATCCTCATAAGTTGTAATAAATAATAAACATTTTTATATCTTCTTGCGTTTGGTATATATAAA CGTAGGGTTGAACAGATGCCGCAAGAGTTGTA
GATTGAGATGGTTGAACTATCTTAAACCAAATATCAAGCGGGGAGATTTCAGTGAAGATGAAATTGATATGATGATCAGATTGCACAAGCTTTTGGGAAA
CAGGTTTGTATATTGGCCATTAATTAAATCACACTACTGAGTGTAGTGATACATATTTAGAAATTAATATGCCTTATATGAGATATTTGAATCCATATTA
TATTATATGCATG*ATAATACAGCTTGGGGTGTACTATTAACTTTTTCCAATATTTAAACCAAAAAGCTAGTTGGTTTTGTTTATGCTAAGAGTTATTTAG
AGATTCAAAGAAGAACAACAAAACAAATCTTTGTTTTCATATATATATATATGGAATACAATTTCACTTTTGGGAACTATTTTTTTACATGAGAATAATT
AATAATAATTATTAGATTAAAATTAAATTAAAATATAATATAATAATTAAATCTCTAAAAGATTTATCAAACAAATGATATCTTAAAATATCATTAATTT
ATCATCATTGTCATAACTGTTATCATCACCGTCATTATGACTGTTGTCGTTGTCACTGTCACCACCATTGTTGGATGATAGCGACGATGATGACAACAAT

**🡨 R6990RPB (37 bp)**
CATGACGATGGTGGTGGTGACAGAGGTGAAAATCATAATGATTTTTGCAATGTTAGTAACTACTGCAATAATGAAGGTCATGACAGTAGAGGTGGTGATA
GTTGACGACGATGATGGTGATAACAGCGACGATTATGGTGGTGACTACAGCAGTGCTCATGGTGGTGACAACGACAATTATAATTATGATTAGTTAATTG
AAATATTCTAGGAAGTGATAATTAAAATCTTTTTAGATTTATTTATTGTTTGATTAATTTTTTAGAGATTTGATGATATATTAAATTTTAATTTAAATTT
TAAATATTTTAAAATCAATTGTTTATTATTTTTAATTGATGATTATTATTAATTGTTCTCATATAAGATAATGTTCTCACAAAATTATATATATATATAT
ATATATATATATATATATATATATATATATATATATATATATAGACATGGACAGCATAAAGGTTTTATACGATGTTATTTAATTAATTAGATTGTCATTT

**🡨 R6990RP1 (31 bp)**
ATAACATTGTTTGAGTTTTGTGGTAATTACTCTAAAAGTTGCATGTAAAATGATAGAGTACATCAAAATTAAAAGAAAAGTGATTAATGAACTTTTCATT
TTTCAATAGTATTACTTCGTTAAACAAAAATTGTTTGTCGATGTAGATGGTCCCTGATTGCAGGAAGACTTCCGGGAAGAACCTCAAACGATGTAAAAAA
TTATTGGAACACCTACGCACGCCGTAAATTACACTCTCACAAGAAAGACAACAACATAGAAAAGCAAGCTAGGGCCAAAACAACCGTGAAACCCCACGAA
GTTATAAAGCCTGTACCTCGAGCTTTAACAAAAACATCCCCACGGTTGCAAGGGAAATTCATTAATAGTTCAGAAGTTGGTGTTAGTCATGAAGAAGGTG
CAACTTCAATATCAGGGTCTGGGAATTGGTGGGAAACTTTTTTAGATGACAAGGAAGACATTGAAGAAGGTAACAACAACAAATGCTTCTTTGGTGGGGA
AGATGGAGCACTTGACCTTTGGGGTGAAGAGCTTAATTCAATTGCTTGTGACTTTCTTACACAAGGTGAAACTTGGAGCGATTTTCTTCTTGACCTAGGG
CTAGGAGATTAGTGTGTGGTGTTTGTTTTCACAAGGGACCTCAAATTCTAATATGCAAGTACAAATCAAACTTACCTAACATTTGAGTTTCCTTTGTAAA
ATATATATATTCTTTACTTTGTATTTGTGAATGTATCTATCTTATCAGTATGTTAAAAAAAAAATTATCTGCTCAGTTATACAGAATGTAAGATTATATC

**Three exons; Two introns**

***site of *TgmR** insertion.**

**PCR Products:**

**R6990FP1** (37 bp) **- R6990RP1** (31 bp) 🡪 **1,451 bp**

**R6990FP1** (37 bp) **- R6990RPB** (37 bp) 🡪 **929 bp**
