# Supplementary material for: Methylation Affects Transposition and Splicing of a Large CACTA Transposon from a MYB Transcription Factor Regulating Anthocyanin Synthase Genes in Soybean Seed Coats
Source: PLoS One. 2014 Nov 4;9(11):e111959. doi: 10.1371/journal.pone.0111959 (PMC4219821; doi:10.1371/journal.pone.0111959)
Supplement: File S2 — TgmR* DNA Sequence with Highlighted ORFs and Terminal End Repeats. (DOCX) [file pone.0111959.s004.docx]

**File S2**

***TgmR^*^*: 13,024 bp**

CACTACTAGAAAATAAGGTTTTAACATCGGTTATTTAAGACTTTCAACATCGGTTATTAATTGATGTTGAAAGTACCGATGTGGAAAGTAGTATCATTAACATCGGTTTTTCAAAACCGATGTTAACTAATAAATACAACATCGGTTATTTAAATAAGCGATGTTATATGATACGAATTATGAAAAAAAATTATAAATCTATAAATTAACATCGGtTTTTTAAAAAACTGAT**GTTGTAAGTGACATTTAACATCGGTTTTTTAAAAACCGATGTTGTAAGTGACATTTAACATCGGtTTTTTaAAAAACTGATGTTGTAAGTGACATTTAACATCGGTTTTTTAAAAAACCGAT**GTTGTAAGTGACATTTAAT**GTTGTAAGTAACATTTAACATCGGtTTTTTaAAAAACTGAt**TTTGTAAGTGACATTTAACATCGGtTTTTTAAATAACCAAT**GTTAAATGTGACATGTGACATCGGtTTTTTAAAAACTGATGTTGTAAGTAACATTTCACATCAGtTTTTAAAAATCTGATGTTGTAAGTGATATTTAACATCGGTTATTTAAATAGGCGAT**GTTATATGATATGAATTATGAAGaAAAAAA**GTTATAAATCTATAAATCAACATCGAtTTTTaAAAAACTGAT**GTT**GTTAGTGACATGTAACATCGGTTTTTAAAATAACCGATGTTAAATGTGATATATGACATCGGTTTTTAAAAAACTGATGTTGTAAGTGACATTTCACATCAGtTTTTTTAAAAACTGATGTGAAATGTCACCTACAACATCAGtTTTTTTaAAAACCGATGTTAAAAAATGTTGAGGTAGGTGACATTTAACATCAGTTTTTaAAAAAACTGATGTTGTAAGTAACATTTCACATCAGtTTTTTTAAAGACCGAT**GTTGTTTTAGAAATTTATTTTTAACATGATGTCTTTTTTTCAATAAATCCCAAAAATAACCTGCAAATTTTAAAATCAGACCACACAACATATAATTTTCATTCTGTTTTGAAACAGTTTTTACCAGAAAATTCAATAAGAAATTTACTAATTACTATGAATTTAAAACATATTTAATGTTGAGACATGAATTGTAAATTAAGTAAGTAAATATAAACTACAATTACAAGATTGTCTAAACATCCTAAGTTTCATTTTTCACTTTCAAATAGTACTTTGCCCACTGGTTGCGAAGTGCCTTCAATCTTTCTGGTTCCAATGGTCTAGGATCAGTGAAATACTGCATGATATAATAAAACATAAGTTAATAATATATTAGCAATCATAATAATATGAAATTTGGTGAATTAAAAATTACCATTTCCCAATTATTCTGGAAATTTCCTAAGATTATAGTTGACATCCAATGCATGACGTAATACCCACACTCAGTGCTTCCTTTTTGTCTATTACACTAAATACATTATGAAATTTAGATATGCAACGTTCAGTACAAATTAGTCTACACAATACTAAAATATAAGTGAAAACATATTGTTTAAATAACTTACTTTAACAACAATCCACCTAGCAGGAGTCTTGGATTTACTTTGTTGAGTATCGTCAAGTCCTTTCAAAGCACTATTGAATACAAACATAGATTCTTATTGTACATTTAAAATTTTGATTTACCTGACTAATGCTAATGCAAATATATTAAAAAACAACACTGACCTATTAATTATGCCTTTGAGGTAGTTGTCTGGCTTATTATGCAACGAACAAAACCAAATGACAACATTTTCCTTAGGCAAAATGACGACCATTTGCCAATGTGCACTGCAGTGGAGACCAATATATGTTATTATACTATTATACATTATTTAAATTCATTTGTTGAGTTTAAGTTACCCATTCAAGTAGGCTCCTAGGTACACATCTCGTTTTGAATTTTGCATCCAGTTCTTAATGTAATTTTCTGATTCAAATTGTGATTGGCCAGATCTCTGGATAGACTGTGGCTCGAGGAATCCATACACATCGATATTCCCAGCTCGCATACTTGTCTCAGTCATATGCCTATTATTGTTAACACAAAGTAAATTATGCATGAAGGTAAAACAATAAATTAGGTAACTAACAATAATCTAAATTGACTTACAGAATCCACAACTGTATAACAGATATGCTGAGACATTGACCACCATGTGCAATTTCAGACAGATCTTCATGCTTTATGTACAAGGGAAAGTTTTCATTAAATAGGCCAAACAAGGTAGCATCCCACATAACCTGCAATGGCTTCAGAAAAAGCTGTGGAATGGTCAATGTCATTAGATATAGCGGATCATCGACCTCATCATCCGGCCTATCTGCAGGTTTCGCGGGTCTCACAGCTCCCTGTTTATCCAACATAATTAATTGACATAATTTAATCACAGTAAACATTTTAATTGGAAAAATAAGCATTTAATGACACTAAAATACCTGTTCTGATAAACGCTTGACTAGATGTGTCGGCCAAGCAAGGAATGTGTTAAGAGCCTGTCCCACGACCTTAACCTCTTTAGTGGGTACAGGAATGGGAGCATCTACATCTCTAATCTCCTCAACACTAACCTTAACTTGATCATGCAGCAAAGGAATGTTGTGAATTGTTGTAGATCCCTCATAAACTCTTCCCAGGGCAACCAGGCGAGAAGGATATTCTTCAATATACAACCCACATTTGTATGAGTCACCGGTGTCTAGATCGTTCCCTGAGGGATCAACACAACTCTCCTTTGTGCTGACACGAGCAGCTGAAGGACCAACATCAGGTTCAGGAGGCAGTGCGAGTCCCTGTGATTGCATTTGGCTGAAGGATAATATTAGCCGTCGAGTGACTTTTTCTGTGATTGAATCCTCTAGTTGGTCCTTGATTTGTTGCGTCAACTGTTGCAGGTATTCGGGAGCAATGGAGGAGGTCCTTGAAGCTGATCCAAAGTATTGTTTGATGGTTATACCGGCTCCTACAGCACGCACACGACCAGGGTGTTCTGGTCGCCCAATGGCAGCAGTCAGTATATCATGACGTCCATGGGTAACAAAGGAACCCTGTGAGGCCTGCTCCTCAAGCGCATCCTGTGAAGAACACATTTATTCTATACTTAATCACACAATAAAAAAATAATTACAATTATGAATTGAAAGTGACTTACAATCTTGTCAGCAATTTCCTTTGCTGCTTCAGATGTCATGTCACCAGTTTTCTTGGTGCGGGCTAGCTTCCACTTCACGTGTCGTTTGATGGGAGATGGAGGATCAATGACGGTGTCAGTGCTTCCGGATTGAGTTGCTTCCTCTAGtTTTTTCTTTCTCTTCTCATCCATCAACtTTTTTTCTAAATATTCATAAcCCCCACGAGACATCACGTGAGGGGCAGTGTTTTGTTTTTGGACAGCTTGTGCTTTTtTCGAACATTCTGTaAAAATGAaACATTAATGAAACTCATGATTACAATGTATTATAATAAGTTCATTTAAAGTTAAAAAATGAAAATCCCAAATTAGTTACCTCCCATGAAGGGTCTCTACGGCTCTGGCAAAATTGGGTCCATTTCTCCTTGCTAATGCCGTACATTTTGCATACAGTGTCATCAACACTATCCTTGTCAGCTGCAAGTGCCCATTTCGACGTCAAATCAGACTTAAACTGTCTCCACCGCTcCCCCACAGTCTGAAGTATTTTCtTTTTTGTCCTTAAATCAGATGCTTCAGGGATATCAAATTCAGCCTAACAAAGAGTAAATTAGGTTTTATTAATACTAAATTCAAATATTTGGCTAACAAACAATATGGAACATGAAATAATACATGATACCTGAATATCCTCCCATATCAAATCCTTCTGAGTAATAGGGACATGCTTCCAATTTTCGTATGTGACATCCACCTTATCACGAGCAACGATCCCTAAATATGTTCTAAATTTCTTGCTGTGGGGACCGTCTGCTTTGCCAGTAACAGGATCCACATGGACAAGGGGTCTCTCTGCCCCAACTGGTCTAGTCGCCAATAATCTTAGGCGTGAGGCCTTTCTAGTCCGCTTCAATGGTGGTGACGACGAATGCGATGTTACACCAGTAGAAGGAGGAGGAGGCGGAGGAGAGTTTGACTGTGTAGCCATTTGGCTGTGAaAAAAaACATTAATTCAATATGTTATaAAAAATAAATGCGTATGAATGTAATTAAATGAATTGAAATTAAGTACAAAATAAAAAAAATTACATTAGACGATGTTAATTAATTCTCCTTCATCGTGATCATTACGATTAGCATGAACATCGTCAGTTTCTTCTCCGATGACATTAGGTGACAATTGTGTGGACAAAGGACTAACATAAGTATCAATGTATGAATAATCATCTTCAACATTGACACCTATTGTTTTTCCATGTAAAACCACTGACCACCTTTGATCACAAGGGTCTTCGACATAAAATACTTGTTTCGCTTGTTCTGCCATAATGAAAGGATCATTCTGGTATGCGAGTTTGTTAAGATCCACCAACGTAAATCCCACATCATCGGTCTGCACACCGGTATTACTGTCAACCCACTTACATTTGAAAACACAGACAGTGAATTTGACATAGTTAAGCTCCCAAATTTCTTCAATGAAACCAAAGTAAGGGATGAAAGCTACACAAGGATTGTCATCATGTACACTAGCGAAGTGTTGAGATTCAGCCCTTAGGGTAACCCCACTGTTTTGCATTGTACtTTTTTGGTCTTGTGCTTTTGTATAGAAGGAATACTTGTTTATATCATATCCTTGCCAAGTTATAACATTTCTTTTAGGTCCATCTGCTAGCTTTCTCAACATTTCAGAAGCATTATCATCAGCCAAGATTGTGTGTTTAAACCAATCTAGGAAAGTCTTGTTATGtTTTTTCAACACTGAGTTCTTCGACAtTTTTGGATTACTTTGTTTGACTAAATTTTCATGACGAACTATGTATGGCAAAACTTCATTACTATTATTCAACACATACAAGTGAGCTTGTTGCAAATCTTCTACACTTGGAGTGATGACATGCAGTCCTCTTGAACCCTTACCTCTTACTCTTTCGTCATGCCGAGACTCGGGAAGCCCAACAGGTTTAGCTTTTTCAATGTACTCTGAACAAAATTCAATGGCTTCTTCTGCAATGTACCTTTCAACAATAGATGCTTCAGGACGGTGTAGATTTTTGGTATACCCTTTTAAGATCTTCATGTATCGCTCAACCGGATACATCCACCGCAAATAAACAGGACCACAACATTTGATTTCTCTGACTAGATGAATAATTAAGTGAACCATGATGTCAAAGAATGCAGGCGGAAAATACATCTCCAACTCACACAATATAATAGCAGCCTCATTTTCCAGTTCGTCTAACTTCACAGGATCAAGAACTTTGCTACATATAGAATTGAAGAAAAAGCACAGCCGAGTTATGGCAAGCCTGACTTTGTTAGGCAAAATGTCTCGTATGGCCACCGATAACAATTGTTGCATCAAGACGTGACAATCATGAGACTTTAAGCCTACCAACTTAAGATCCTTCAAATGCACAAGGCTCTTAATATTTGAAGAGTATCCTTGTGGAACTTTGACCCGTCGTAGGCATTGACAAAAACTGAACTTTTCCTTTCTGGACAAAGTATGACAAGCTGGAGGCAAGTATATTTTTCTACCATCAGACCTTGGATGTAACGACGCTCGAATACCCATCTCAGCTAGATCTTGACGAGTATTCAAACCATCTTTTGTCTTGCCGTGAATGTTAAGGAGCGTCCCAATGAGACTATCACATACATTTTTCTCCACATGCATAACATCAATACAATGTCTAACATCTAGATCAACCCAATACGGAAGATCAAACAAAATCGACCTTTTCTTCCATATGCAAGTCTTAtTTTTGTCTTTCTTTTGGACCTTTCCAAATATTGTATTTAGGTGTTGAACCCGCTGGAA**AATCTGGT**CATCAGTCAACGGTACCGGTGCATTTTCATGCTCTTGACTTCCATTAAATGCtTTTTTCAATCGTCGGTAAGGATGATGAGGTTTAAGAAAACGTCGATGCCTAGTGTAAACTGTtTTTCTTCCATGTTTCAGTTGTATGTAGCTTGTGTCTTCTTCACAGATGGGGCATGCACGATGGCCTTTAACACTGTAACCGCTCAAATTCCCATATGCTGGAAAATCATTAATGGTACaAAAAAGCATTGCACGTAAATTAAATGTCTCATTTCGAAACCCATCAAACACTAAAACTCCTTCGTCCCACAACTTTGTAAGGTCTTCAATCAAGGGATTGAGATAAACATCGATGTCATTTCCTGGCTGTCTTGGGCCCGTTATCATCATAGACAACATCATGTATTTTCGTTTCATACACAACCAAGGAGGCAAATTGTAAATCACTAGCAAAACTGGCCACGAACTGTGTTGCGTGCTTAAACTGCCATATGGATTCATTCCATCAGTGGCTAGTCCAAGCCTAAGATTTCTTGCCTCTTTGCCAAAATCCGGATACAAACGGTCTATCTTCTTCCATTGCAAGGAATCAGCCGGATGACGAAGCATTCCATCACAGTTTCTCTCATTTGCATGCCATGTAAGGTCTTTTGCGTCGTCTCCATTAGCAAACATACGCTTAAACCTTGGAATGATCGGAAGATACCACAACACCTTCGCTgGGGGGCCCTTGTTTGAGTTTTCATCACTACTACACTCCTCATCATCCTTGAGTTTGTACCGTGATACCCCACACCTAGGGCATTTCGACATTTCTTGAAATTGATGTCTGTATAATATGCAATCATTCGGGCAAGCATGAATCTTCTGATACTCCATACCCATGGGACACAATATTTTCTTTGCTTGATAGTAACTTTTAGGCAATGTGTTTTCCTCTGGAAGCATGTCGTGCACTACTTGAAGCAGTGAACTAAAGCTTTTGTCACTCCACCCATATCTGGCTTTGATATTAACCAGACTTAAAACTGCCGACAACAGCGTCAACGAATTCTTGCACCCCGGATACAAAGGTTTCTTTGAATCAGTTTGTAATGTATCATACATAGGGGCATGTGCTTGCTGAAAAGACTCTTGTCCAAGGTCACGAATCATATCCTCCAAGCGATCTCCCATTTCTACATCAAACGGTTCGGATTGTTGCCCACTCTGCATGTCTGTCATTTCACCATGCCATATCCACGTCGTATAATTCCTCTTAATTCCATCACACAACAGATGCTCTCGTATGTCATCCAGTATTTGTCGTCTTCCATTCAAACAATTGATGCAAGGACAATAATATTTTCCATCTTCATCCGGTTGACCTCTTTCTGAAGCAAATTGTAAGAATTGCTCGACGCCTTCCTCATATTCTGGGCTGATGCGACTTTGATTCATCCAACTTCGATCCATCTAAGTAATAACTCGGTCATACTCTCAAAGTTATTCGATGCATGAAAATCTCACTtTTTTATTAAAGGTGTGGCCCTATCCCATTTGGCAAGACCGTCTTTTATGGTAGCTTCATACATCAGGGTTAATTCTATTTTGTTAAATTTGACAAAATTTTGGCAGCATTTCGCATTGGTCTCTAAGTACACGACATGAAATCTGTGAAATTAATTTCCCGATAATCAAGTATGCACTCAGAGAACAACTAGAAATGCATTACCAAAATTTCATCAAATTAAACAAAATAATTTTAACCTTAACACATGAATCTACCATAAAAATATCAGTCTCCCCAAACTGTCCAAACGGACAATTCAAGATTGAATACAATGATTAATGCAAACTGTCCGCAAGAATCATTGCATCCAATCTCAAACGGGAATCTAAGGTTCACTCATCATGAACACAATTTGTATAGCATATATCAATTGTCATTAATGGCAATGAACACGTAATAAAAATATTCATTGGTAACAAACATTTGTACCTGTGATGATGAGCAGCACGGTCCAAAATGAAAACAAGAATCAAAGAAATAACTGAATGAACACCTACATAAGACAATCTTGTGTATGATAAAAAAAAaAtGAGTAAGATTAAGTATAACAAGCGCTCAGAAGATACGAGAATAATTTATTCTAAATTATACACATAACTTCTATTACCTCTCACAAGATCACACGAGAGATATGTACAACTTCTAAAAAGTAGTTGCTGAATCTTATATGTATAATTAATATGTACCTTAATCCAACGCCTTCTTAACTTTGGTCTTGAACAGTAGTATATATAAATTTTCCATTGAAAAATAAAGAAAACACCCCGTTGAATGTGTCACAAATGAGCCAAAATTTGAGCCTACAATGCAATGCCAGACCGGACCATACGCTTTGTCAAATTCCTGCACATGAACATACCAGGAATACATCGTAGATAAATAAATAATTTTATCAACTGTTAAAAAATGGAGAAGAAAGAAGCACTATATTTTATAGATCTAGCTGATCAAATATAAATAAATAAACCAACTA**TAATGTG**AAATTATTTTAATTTAATGAAACTGACGGTGTATCACACGAGATCAAATAATTATGTTTTTTTATAAAAAAAATTATACTTTTTTTATCTCTTTCATTCTTTTTATTTCCTCCTTGCTTATCTATATTTGACAAAGTTTATGAAATAGATGTGGAATTTGGTATAGCGGTGGGCTGCGTATGGATTCTTACGAAATGATAAGTTCAAACTATTCAAATTCATTTTAAGTTTTAGTCTTTAAGCCATAAAACAATAAATTTTAGTCCTTGCACTTACATAAAAAAAGGTTTGGTAACAAGTTTTAGTGACGTAAAAAACTATTACAAAAATCTTTTCACGGAGTTAAATTGGCACaAAAAATTGTTTAAAGACTAtTTTTTTTTTTcAAAAAAAAGACAAGTATATGAATCAATTGAAAGCATAAACAAAATGATAGTCATAAGAACATATGTTGGCAGTTAACCTTAAtTTTtTTTTCAGAGCACAAATATAATTTTTACTATAGATAACTTGTCCGAATGCACAAGATGGACAGCAAAATTTTGATTAAATTAAAACAACTTCACATCAATTAATTAACATGCTTAATAGTTGATAACCTTTATCCTCTTAAAATATATTTAGACTTTCAATTTTCAATATCAGTACTCTAAACGAATCTCAATACTTTTGAGTTTTGATTCATTCTTGACTGAATACTAATTAAGAGCACTAAAAATTTCTTAGAATGCAGTATTTTCATTATTTCATCATTTAtTTTTTTCTACGTTTTTTATCATCTGTCATCGACATCCATTGAAAGATTTGTAAGCTTTAATGACCATAGTTAATTTCCTTTTCCGTCTTACCTAGTTTAGTTTTTATTCAAAATTTTGAGAATAGAACGATTCTCAAACACGTCTTTAATTAAAGAACATTTGCTTTGCAAAAAATAATTATTAGCAATGAACATAATTAATTTTAAAAAaACAAATAATTAAGCCTTGTCTGTTCACTGTTTTCAAACTTCAAAGAGCAAGAAATAAGCGGAATGCAGAGCAATTCATCAATGCCACCTCCGAACTCAAAATCGAGACCTAAAATTAATCAAACGGACTTCGAAATGAGTTAACCACTCGGAGACGAAGCAAAACCTTAAGCTCAAGACTCTAACaAAAACAACAATAACAACCTGCATTTAGAGAGGTATTTGCCCCAACCACAAAAAAAAGAAGCAAAGTTCAATAAGAGAGCCAAACAAAAGAGACGCAAACCTGATTGCGGTGGCGTCTCAGAGGTGTGCGGCTGCGCCGTGGTGCTGATTGCTGCAAGGGTTTTGCGTCCCAGCGCAATGCAGAGAAGCTTCTCTGCCCGATGCGAACTCCAAGAGCGAAGGGAACCCTGTCCTCCGCCACAGGTCGCTGCCGTCGATGAAGCACCCACCGTGTGCAGACCGTCGACGCTTCTCTTTTGCCGAGTGAGTGACTGAGAAAGGGTTTTGTGTGTGCCGCCGAGTGAGTGAGGAGTGATGTGTGCGCTTTAGCTATTAAAAAACAAATTACAGTAACCTTTAAATTGAAGGTGTGAATTATTATTATTATTATTATATTTTGAAACTATTTTAGCAAAAATATAAAATTGTTTGACTCGTATTTCATCGAGCAATTTGCTAAAGAGGACGTTTGATAGGAGAAAGAtTTTTTGTTTTTTAGAATCATAGGTTAAAAAATTTAGGTGGTGTTTTGTTTGATTTGCTTCTGTTTTTATTTTTTTAAAAATAGAATATAAGAGTaAAAATATTTTTGTTTCAAAtTTTTTaAAAAATATTTTATCGaAAAaTATTTTaAAAAATAGAATCAAAATTaAAAACaACAAATGTTTTTAATTTTTCTAAATAAGTGAAAATATGATAACACTTTAGAGTAtTTTTTtgttgataaatattttttaaatcttgaaaatttaaaaataaaaattattttcaaaaaatgaaaataaaaaataaaaaacagaaaatAAACACTCAAaTcAaGaCTCAtCTTAGaTTTTtGATGTTtGATAGGtAcTTTTAAAAATAAAATTTACGaGAAAAGATATTTtGTGaagatGTTTttAaTCAATTTTTCACGAAaTTTTTTtCCAtGgAGAGAGTTAGAAATTTTATTTTTtCAGTTtAATTTTTTtATTATAATAAAAAAaTTATATTTTTTATtAAATtATATAATATGGTAAaTAATTTAAATAATTAGTAAAAaTATGGATTCTTAGAAAaTTTATCTAATTTTTTtAATAATCAATCAAaTAAATTTGATTGATTCAAAAAAaTATAATTTTCATGTGaTGATTATTAAAAATTATGATATCGAACATGAAAAACTTCTTTACTACTGAATGTCCCTAAAAGTAaTTTTTTttCTTTTTtCTATAGTCCTTCGTTCTCAAAACTACCATCTTTTATTGTACCATATGAATGGAAATTACAATGAAAATGTTACATTTAAAaTTATTGACATGAAGGAAAGGACTCTTACTTGAGTAGCGTCCTTAAAAaTAAATACAAATATAATTTTTATTTAATTgATAAATATTAATTATtAATTTATTAATTTTtGTTAGTGAAAAATTTAAATTCACAACCTATCTCTTCTTTTCTCTCTTTtATTAATCTTTTtGGTCTCAATGGTGTGAAACTAATTTTTTTtAATTATTATAATTTATATTTTAATTCTCTTTtAATTTTTATAAAAATTAAAAAaTATATTTTTAGAGAAGTAAAATGTAAATTATAAAAaTTATAAAAaCCAAATAAGTAATTTAAATTTTTTAAGTGATTCAAATTTTAGGGTTATTAAAAAaCTACAAATCCATGAGGTTTCTGATCGCATTCTTACTGCTGAAAGTCTTAATTAACCAATTAGTCTTCAGTCTGTTGGAAGCGTTCAATCCTGTGAAGCAACAAACTGTTTGCATAATCCTGTGGTGTATTTGACATCGTAGCAATCAATGGATATGGGAAAACACAAATACGCAGCCATCAACTTTAGTCCACTCGGAATGGTCGAATGAATGCCCCCACAAATCACTATTTTCTTTAAAGTAGAAAATCCTTTCTCAATTTACGTTTTAAGCTTCATTTTATGTTAGGCTGACATTGAATTGGTGCTCATAGAATATTAATTAAAAAaTTTAAAAAAAaTTATTATATAAATCACAAGAGATTATAAATACTTATCAGTATAATTTTTATTTAATATTATTGAATACTACTACCAGTATAATTTTTATTTAATTTTATTATAAATACTTATCAATCAGTTGTTTTTAAGTGTATTTTCGGTTTAGCATCTCCTAATGTAAAATCCACGATTGCAGTGAAACTGTTAATTGATTTTATTTTTTTtAATTTTTAACCAATTGACGTAGTATTTTATGTTCTTTAACGTTTTTTCTAAAATATGTATCTTTTtAACGGCATGTATTTAATATTATGCAAGAATCATATCTTAAATTAATTTTTAGAATTTTTTATAAATAACAATGATTTTATATTTATTCTGAAATATGTCCATTGAAATAAATTATTTTATCGGTCATAGAGATCTGACTAAACAATTATTTTTTTGTCTTTtCTTGGTTGAATAGTTATTATATATTTTTATGTTTAATTTTTACGAATAAAAAAaTTAATCAATCCGACCCTACACTTACTTGGATAGTAGTCACACACTACATTTGGACATTTGCAGTAGATCGATCTTTGACAAAAAAaGTTACGCAAGATTTTAATTTCTATAAAGAAATCATATAATTTTTTtATATCATTCATTAGTTAAAATTTATTATTACTACAATTTTTAGGATAACTAACATGAAAAAAATTGTACATAATAATTTATTATTAAATATTAGTGTAAAAAaTAAAATTATTAACATATACTATTTACTCTTTTTTTAGTAGCATCCACTATTTACTCTTAACACAATGCATTTGGTAATAACCAAAATATTAAATTTTACGTCCATTTGTATGTTG**GTTAAATGTCACTTACAACATCAGTTTTTTAAAAAaCCGATGTTAAATGTCACTTACAACATCAGTTTTTtAAAAAaCCGATGTTAAATGTCACTTACAACATCAGTTTTTtAAAAAaCCGATGTTAACTAATGATGTTAACATCGATTTTCCAAAAAaCcgaTGTTAACGTGTATGCATTAACATCGGTTTTTtGGAAAaCCGATGTTAACATATTACACGTTAACATCAGTTTTTGAAAAACCGATGTTAACGTGTATGCATTAACATCGGTTTTTGGAAAaCCGATGTTAACATATTATACGTTAACATCGGTTTTTGAAAAACCGATGTTAACAAATGATGTTAACATCGGTTTTCCAAAAAaCGATGTTAACATCATTAGTTAACATCGGTTTTTtGGAAAaCCGAT**GTTAAGAATGATACTTTATTTACAAATATGCCACCGCGTTTAACTTAACATCGGTTTTGTAAAAAACCGATGTTAATAAGCCGATGTTAAAACTACTTTTTGTAATAGTGATG

>Glyma05g23600 Homology

| [**Pfam:**02902](http://pfam.sanger.ac.uk/family?type=Family&entry=PF02902) | Ulp1 protease family, C-terminal catalytic domain |
| --- | --- |
| [**Pfam:**02992](http://pfam.sanger.ac.uk/family?type=Family&entry=PF02992) | Transposase family tnp2 |
| [**Panther:**10775](http://www.pantherdb.org/panther/family.do?clsAccession=PTHR10775) | AC-LIKE TRANSPOSASE-RELATED |
| [**Panther:**10775:SF11](http://www.pantherdb.org/panther/family.do?clsAccession=PTHR10775:SF11) | AC-LIKE TRANSPOSASE |
| [**GO:**0006508](http://www.ebi.ac.uk/ego/DisplayGoTerm?id=GO:0006508) | proteolysis and peptidolysis |
| [**GO:**0008234](http://www.ebi.ac.uk/ego/DisplayGoTerm?id=GO:0008234) | cysteine-type peptidase activity |

Glyma15g21480 Homology beyond the green highlight.

| [**Pfam:**02992](http://pfam.sanger.ac.uk/family?type=Family&entry=PF02992) | Transposase family tnp2 |
| --- | --- |
| [**Panther:**10775:SF11](http://www.pantherdb.org/panther/family.do?clsAccession=PTHR10775:SF11) | AC-LIKE TRANSPOSASE |
| [**Panther:**10775](http://www.pantherdb.org/panther/family.do?clsAccession=PTHR10775) | AC-LIKE TRANSPOSASE-RELATED |

**TAATGTG – CCGATGTTAA Homology to Gm08: 93539…98330 Reverse Complement.**

***Tgm1* Homology**

|  |  |
| --- | --- |
|  |  |
