# Supplementary material for: Methylation Affects Transposition and Splicing of a Large CACTA Transposon from a MYB Transcription Factor Regulating Anthocyanin Synthase Genes in Soybean Seed Coats
Source: PLoS One. 2014 Nov 4;9(11):e111959. doi: 10.1371/journal.pone.0111959 (PMC4219821; doi:10.1371/journal.pone.0111959)
Supplement: File S3 — TgmR* Terminal Direct Repeats. (DOCX) [file pone.0111959.s005.docx]

**File S3**

***TgmR** 5’ and 3’end sub-terminal sequences highlighting the direct and invert repeats containing the TNPA and TNPA-like binding domains shown in Figure 3B**

***TgmR** 5’-end direct** (yellow highlight) **and inverted** (green highlight) **repeats**

CACTACTAGAAAATAAGGTTTTAACATCGGTTATTTAAGACTTTC**AACATCGGTT**ATTAATTGATGTTGAAAGTA**CCGATGTG**GAAAGTAGTAT**CATTAACATCGGTTTTTCAAAACCGATGTTAA**CTAATAAATAC**AACATCGGTT**ATTTAAATAAGCGATGTTATATGATACGAATTATGAAAAAAAATTATAAATCTATAA**ATTAACATCGGtTTTTTAAAAAACTGATGTTGTAAGTGACATTTAACATCGGTTTTTTAAAAACCGATGTTGTAAGTGACATTTAACATCGGtTTTTTaAAAAACTGATGTTGTAAGTGACATTTAACATCGGTTTTTTAAAAAACCGATGTT**GTAAGTGACATTTAAT**GTTGTAAGTAACATTTAACATCGGtTTTTTaAAAAACTGAt**TTTGTAAGTGA**CATTTAACATCGGtTTTTT**AAATAACCAAT**GTTAAATGTGACATGTGACATCGGtTTTTTAAAAACTGATGTTGTAAGTAACATTTCACATCAGtTTTTAAAAATCTGATGTTGTAAGTGATATTTAACATCGGTTATTTAAATAGGCGAT**GTTATATGATATGAATTATGAAGaAAAAAA**GTTATAAATCTATAAATCAACATCGAtTTTTaAAAAACTGAT**GTT**GTTAGTGACATGTAACATCGGTTTTTAAAATAACCGATGTTAAATGTGATATATGACATCGGTTTTTAAAAAACTGATGTTGTAAGTGACATTTCACATCAGtTTTTTTAAAAACTGATGTGAAATGTCACCTACAACATCAGtTTTTTTaAAAACCGATGTTAAAAAATGTTGAGGTAGGTGACATTTAACATCAGTTTTTaAAAAAACTGATGTTGTAAGTAACATTTCACATCAGtTTTTTTAAAGACCGAT**GTTGTTTTAGAAATTTATTTTTAACATGATGTCTTTTTTTCAATAAATCCCAAAAATAACCTGCAAATTTTAAAATCAGACCACA

A total of 8 possible loop structures

***TgmR** 3’-end direct** (yellow highlight) **and inverted** (green highlight) **repeats**

**GTTAAATGTCACTTACAACATCAGTTTTTTAAAAAaCCGATGTTAAATGTCACTTACAACATCAGTTTTTtAAAAAaCCGATGTTAAATGTCACTTACAACATCAGTTTTTtAAAAAaCCGATGTTAACTAATGATGTTAACATCGATTTTCCAAAAAaCcgaTGTTAACGTGTATGCATTAACATCGGTTTTTtGGAAAaCCGATGTTAACATATTACACGTTAACATCAGTTTTTGAAAAACCGATGTTAACGTGTATGCATTAACATCGGTTTTTGGAAAaCCGATGTTAACATATTATACGTTAACATCGGTTTTTGAAAAACCGATGTTAACAAATGATGTTAACATCGGTTTTCCAAAAAaCGATGTTAACATCATTAGTTAACATCGGTTTTTtGGAAAaCCGAT**GTTAAGAATGATACTTTATTTACAAATATGCCACCGCGTTTAACTTAACATCGGTTTTGTAAAAAACCGATGTTAATAAGCCGATGTTAAAACTACTTTTTGTAATAGTGATG

A total of 10 possible loop structures

Highlighted in blue are nucleotide mismatches

Highlighted fuchsia are the CACTA ends.
