# Supplementary material for: Methylation Affects Transposition and Splicing of a Large CACTA Transposon from a MYB Transcription Factor Regulating Anthocyanin Synthase Genes in Soybean Seed Coats
Source: PLoS One. 2014 Nov 4;9(11):e111959. doi: 10.1371/journal.pone.0111959 (PMC4219821; doi:10.1371/journal.pone.0111959)
Supplement: Table S1 — Summary of Sequence Reads from 454, Genomic DNA, Methylation and RNA-Seq Sequenced Libraries Made from RM30-R*, RM55-rm, and RM38-r Soybean Lines. (DOCX) [file pone.0111959.s006.docx]

**Table S1**. 454, RNAseq and Genomic DNA sequence reads for each of two 14 kb PCR fragments and multiple RNA and genomic DNA libraries from the two, RM30-*R** (black- seeded) and RM38-*r* (brown-seeded), lines or the variegated seeded line RM55-*r^m^* (brown/black striped seeds).

454 sequence reads for each of two PCR fragments containing the *TgmR** transposon insertion

| **Sample** | **Internal ID** | **HQ* Reads** | **Average Read Length** |
| --- | --- | --- | --- |
| RM30-14kb DNA fragment | GM04--‐001--‐11r | 24,848 | 507 |
| RM55-14kb DNA fragment | GM04--‐002--‐12r | 13,321 | 507 |
| *HQ= High quality. | | |  |

| **Sample ID** | **Sequence_filename** sampleID_index_lane#_read#_001.fastq | **Read_counts** |
| --- | --- | --- |
| GZRM30 | GZRM30_GCCAAT_L00M_R1_001.fastq | 224,411,040 |
|  | GZRM30_GCCAAT_L00M_R2_001.fastq | 224,411,040 |
|  |  |  |
| GZRM38 | GZRM38_CAGATC_L00M_R1_001.fastq | 195,476,736 |
|  | GZRM38_CAGATC_L00M_R2_001.fastq | 195,476,736 |
|  |  |  |
| GZRM55 | GZRM55_CTTGTA_L00M_R1_001.fastq | 198,361,408 |
|  | GZRM55_CTTGTA_L00M_R2_001.fastq | 198,361,408 |

Genomic DNA sequence reads for each of three shoot-tip DNA libraries from three isolines:

RM30 (black seed), RM38 (brown seed) and RM55 (variegated)

Bisulfite sequenced DNA libraries from RM30 and RM55 soybean lines with the *TgmR** insertion

in Glyma09g36983 Intron2

| **Sample** | **Name of Fastq** (sampleID_index_lane#_read#_001.fastq) | **Number of Reads** |
| --- | --- | --- |
| GZRM30 | GZRM30_CGATGT_L002_R1_001.fastq | 104,639,779 |
|  | GZRM30_CGATGT_L002_R2_001.fastq | 104,639,779 |
|  |  |  |
| GZRM55 | GZRM55_TGACCA_L003_R1_001.fastq | 113,000,270 |
|  | GZRM55_TGACCA_L003_R2_001.fastq | 113,000,270 |
|  |  |  |
| UC44 | UC44_CAGATC_L006_R1_001.fastq | 117,494,482 |
|  | UC44_CAGATC_L006_R2_001.fastq | 117,494,482 |

RNAsequence reads for each of eight libraries seed coat samples of two lines, RM30 (black seed)

and RM38 (brown seed) at five stag

| **Sample ID** | **Seed developmental stage** | **Read_counts** | |
| --- | --- | --- | --- |
| R42_RM30_100.fastq | 100-200 mg fwt green seed coats | 56,829,223 | |
| R43_RM38_100.fastq | 100-200 mg fwt green seed coats | 56,448,267 | |
| R122_RM30_200.fastq | 200-300 mg fwt green seed coats | 50,499,616 | |
| R124_RM38_200.fastq | 200-300 mg fwt green seed coats | 77,784,853 | |
| R123_RM30_300.fastq | 300-400 mg fwt green seed coats | 32,054,833 | |
| R125_RM38_300.fastq | 300-400 mg fwt green seed coats | 30,497,284 | |
| R111_RM30_400.fastq | 400-500 mg fwt green seed coats | 57,883,771 | |
| R113_RM38_400.fastq | 400-500 mg fwt green seed coats | 47,669,711 | |
| R110_RM30_300.fastq | 300-400 mg fwt red seed coats; d* | 38,909,392 | |
| R112_RM38_300.fastq | 300-400 mg fwt yellow seed coats; d* | 39,154,834 | |
| R154_RM30_300.fastq | 300-400 mg fwt green seed coats | 49,461,147 | |
| R156_RM38_300.fastq | 300-400 mg fwt green seed coats | 41,304,290 | |
| R155_RM30_400.fastq | 400-500 mg fwt green seed coats | 44,092,473 | |
| R157_RM38_400.fastq | 400-500 mg fwt green seed coats | 45,475,312 | |
| d*= late seed developmental stage starting desiccation | |  |  |
